# The deubiquitinase USP15 drives malignant progression of gastric cancer through glucose metabolism remodeling

**DOI:** 10.1186/s13046-024-03152-2

**Published:** 2024-08-20

**Authors:** Longtao Huangfu, Huanbo Zhu, Gangjian Wang, Junbing Chen, Yongqi Wang, Biao Fan, Xiaoyang Wang, Qian Yao, Ting Guo, Jing Han, Ying Hu, Hong Du, Xiaomei Li, Jiafu Ji, Xiaofang Xing

**Affiliations:** 1 Key Laboratory of Carcinogenesis and Translational Research (Ministry of Education), Division of Gastrointestinal Cancer Translational Research Laboratory, Peking University Cancer Hospital & Institute, Fu-Cheng Road, Beijing, 100142 China; 2 Gastrointestinal Cancer Center, Peking University Cancer Hospital & Institute, Beijing, Fu-Cheng Road, Beijing, 100142 China; 3 Department of Pharmacy, Peking University Cancer Hospital & Institute, Fu-Cheng Road, Beijing, 100142 China; 4 Department of Pathology, Peking University Cancer Hospital & Institute, Fu-Cheng Road, Beijing, 100142 China; 5 Biological Sample Bank, Peking University Cancer Hospital & Institute, Fu-Cheng Road, Beijing, 100142 China; 6 State Key Laboratory of Holistic Integrative Management of Gastrointestinal Cancers, Beijing Key Laboratory of Carcinogenesis and Translational Research, Peking University Cancer Hospital & Institute, Fu-Cheng Road, Beijing, 100142 China

**Keywords:** Ubiquitin specific peptidase 15, Glucose metabolism, Hexokinase domain containing 1, Insulin like growth factor 2 mRNA binding protein 3, Gastric cancer

## Abstract

**Background:**

Ubiquitin-specific protease 15 (USP15) exhibits amplifications in various tumors, including gastric cancer (GC), yet its biological function and mechanisms in GC progression remain elusive.

**Methods:**

Here, we established stable USP15 knockdown or overexpression GC cell lines and explored the potential mechanism of USP15 in GC. Besides, we also identified interacting targets of USP15.

**Results:**

USP15 knockdown significantly impeded cell proliferation, invasion, epithelial-mesenchymal transition, and distal colonization in xenograft models, while enhancing oxaliplatin's antitumor effect. USP15 was involved in ubiquitination modification of glycolytic regulators. Silencing of USP15 suppressed glycolytic activity and impaired mitochondrial functions. Interference with USP15 expression reversed tumor progression and distal colonization in vivo. HKDC1 and IGF2BP3 were found as core interacting targets of USP15, and HKDC1 was identified as a substrate for ubiquitination modification by USP15, whereby USP15 regulated glucose metabolism activity by inhibiting the ubiquitination degradation of HKDC1.

**Conclusions:**

Our study unveiled aberrantly high expression of USP15 in GC tissues, correlating with malignant progression and nonresponse to neoadjuvant therapy. USP15 inhibitors, if developed, could be effective in promoting chemotherapy through glucose metabolism remodeling.

**Supplementary Information:**

The online version contains supplementary material available at 10.1186/s13046-024-03152-2.

## Introduction

Gastric cancer (GC) is one of the most common malignant tumors in the world, with the third highest incidence and mortality rate in developing countries [1]. According to the Global Cancer Observatory 2022 statistics [2], there were about 478,000 new cases of GC and 374,000 deaths in China alone, which made the prevention and control of GC a big challenge. Due to the lack of typical symptoms in early stage, most patients are diagnosed with advanced stage of GC and misses the best opportunity for traditional treatment [3]. Currently, there is a clinical shift to neoadjuvant therapy (NAT) to achieve tumor downstaging and suppress metastases, and result in facilitating the subsequent surgery and local chemoradiotherapy. However, the popularization of NAT for GC has not been smooth in clinical practice, which are mainly caused by limited number of therapeutic drugs, the diversity of patients, and the adverse effects of toxic side effects [4]. Therefore, there is an urgent need to expand the development pathways of therapeutic drugs and to find new targets for GC.

Abnormalities in the ubiquitination modification system are closely associated with the occurrence and progression of malignant tumors [5]. Recently, increasing evidence has shown that ubiquitin-specific proteases (USPs), the largest deubiquitinase subfamily, play an important role in GC. For instance, USP3, USP5, USP13, USP15, USP21, USP22 and USP35 are upregulated in GC and can be used as independent prognostic markers in GC patients [6, 7]. USP15, one of the most important members of the USP family, has been found to have some amplifications in many tumors, including GC [8]. USP15 was found corelated with E3 ligase TRIM21 and controlled ASCL4 stability to maintain imatinib sensitive/resistant status of gastrointestinal stromal tumors [9]. In our previous study, we demonstrated that USP15 expression in GC is regulated by the miR-26a signaling axis, which may be the main reason for the abnormally high expression of USP15 [10]. However, the downstream function and core mechanism of USP15 in GC is not clear.

With tumor progression, the cancerous cells undergo comprehensive metabolic reprogramming [11]. Aerobic glycolysis, also known Warburg effect, is the most important feature of tumor metabolism [12]. Unlike oxidative phosphorylation (OXPHOS) in normal cellular mitochondrial for energy supply, tumor cells preferentially obtain energy through the glycolytic pathway even when oxygen levels are sufficient [13]. Notably, despite the significant enhancement of glycolytic activity in tumor cells, the OXPHOS process remains intact and functional, and the two coordinate with each other to achieve metabolic symbiosis that drives malignant progression [14]. Therefore, targeting enzymes that regulate the initiation phase of glucose metabolism will lead to an attractive new therapeutic strategy for reversing tumor progression.

Here, we identify USP15 as a deubiquitinase that enhances glycolytic activity and maintains stable mitochondrial function, at least by regulating hexokinase domain containing 1 (HKDC1) and insulin like growth factor 2 mRNA binding protein 3 (IGF2BP3).

## Methods

### Clinical specimen collection

282 GC tissue samples and 162 pairs of GC tissue samples and adjacent normal tissue samples were obtained from GC patients who underwent surgical treatment without preoperative radiotherapy and/or chemotherapy at the Peking University Cancer Hospital. The specimens were snap-frozen in liquid nitrogen and stored at − 80 °C until use. The histology and age information are included in Supplementary Table S1. The tumor-node-metastasis (TNM) stage of GC was determined in accordance with the Cancer Staging Manual of the American Joint Committee on Cancer (8th edition). All patients involved in this study have written informed consent, in compliance with the Declaration of Helsinki. The Ethics Committee of the Peking University Beijing Cancer Hospital has approved this clinical specimen study (Permission number 2019KT111).

### Cell cultures

The GC-derived cell lines SGC7901, BGC823, NUGC3, HGC27, AGS, MKN45, MGC803, MKN28, NCI-N87, and the normal gastric mucosa-derived cell line GES-1 were purchased from the Chinese National Infrastructure of Cell Line Resource (Chinese Academy of Sciences, Shanghai, China). All cell lines, which were obtained between 2014 and 2015 and authenticated by short tandem repeat profiling, were cultured in Dulbecco’s modified Eagle’s medium (DMEM) (Gibco; Invitrogen Corporation, Carlsbad, CA, USA) supplemented with 10% fetal bovine serum (Gibco) and maintained at 37 °C in 5% CO_2_ as previously described. Cells were incubated in serum-free medium and treated with cycloheximide (CHX, Sigma-Aldrich Pty. Ltd., Merck KGaA, Darmstadt, Germany) or MG132 (Selleck Chemicals, Shanghai, China). Routine testing for Mycoplasma contamination was performed using polymerase chain reaction (PCR). Cells were grown for no more than 20 passages prior to experimentation.

### Establishment of stable USP15 knockdown or overexpression cell lines

The shRNAs of human USP15 lentivirus vector were obtained from GenePharma (Suzhou, China). Using a three-plasmid transient co-transfection method (Lenti-T HT packaging mix), replication-defective vesicular stomatitis virus-G-pseudotyped viral particles were packaged in HEK293T cells. Lentivirus-containing supernatant was harvest at 48 h post-transfection, purified by centrifugation and stored at − 80 °C. For viral transductions, 1 mL of the scrambled shNC or shUSP15 lentiviruses were incubated with SGC7901 and BGC823 cells overnight at 37 °C in the cell culture incubator. Stable knockdown of USP15 were selected with puromycin (0.8 μg/mL) in the culture media. After the establishment of stable knockdown cell lines, we preserved these cell lines in liquid nitrogen. All experiments in this research were performed in 3–6 generations of these cell lines to ensure the knockdown efficiency.

The full length of human USP15 was cloned into pcDNA3.1 ( +) vector to generate pcDNA3.1-USP15-Flag vector. NCI-N87 cells were seeded in six-well plates at 70% confluence before transfection. Transfections were performed using Lipofectamine 2000 according to the manufacturer’s instructions. After 48 h post-transfection, puromycin was added at a concentration of 0.8 mg/mL to establish stable cell line of NCI-N87 with Flag-tagged USP15 ectopic expression.

### RNA sequencing analysis

Total RNA from the SGC7901 cells with USP15 knockdown and negative control were isolated using RNeasy mini kit (Qiagen, Germany). Paired-end libraries were synthesized by using the TruSeq RNA Sample Preparation Kit (Illumina, USA) following TruSeq RNA Sample Preparation Guide. Briefly, the poly-A containing mRNA molecules were purified using poly-T oligo-attached magnetic beads. Purified libraries were quantified by Qubit 2.0 Fluorometer (Life Technologies, USA) and validated by Agilent 2100 bioanalyzer (Agilent Technologies, USA) to confirm the insert size and calculate the mole concentration. Cluster was generated by cBot with the library diluted to 10 pM and then were sequenced on the Illumina HiSeq X-ten (Illumina, USA). The library construction and sequencing were performed at Shanghai Biotechnology Corporation. STAR (version:2.7.6a) was used to map the cleaned reads to the human GRCh38 reference genome with two mismatches 1. Then, we ran Subread/featureCounts (version:2.0.2) with a reference annotation to generate gene expression counts and TPM values for known gene model. All the sequencing data have been deposited at the NGDC database with the accession number: HRA005931.

### In vivo tumorigenicity and metastasis assays

Female BALB/c nude mice (Five-weeks old) were purchased from Beijing Vital River Laboratory Animal Technology Co. Ltd. All animal experiments were conducted in accordance with the Institutional Animal Care and Use Committee guidelines at Peking University Beijing Cancer Hospital (Permission number EAEC 2019–03). We implemented the "3Rs" principle of Replace, Reduce, and Refine, advocated experimental animal welfare, and used the smallest statistically significant sample size in this study. Subcutaneous xenograft tumor model was used to estimate the effects of USP15 knockdown on tumorigenicity in vivo. The mice were randomly divided into four groups (*n* = 5): sh-1# + saline, sh-NC + saline, and sh-NC + L-OHP, sh-1# + L-OHP. Each 2 × 10^6^ USP15 sh-1# or sh-NC SGC7901 cells were resuspended in PBS with 50% Matrigel and engrafted subcutaneously into flanks of nude mice. The tumors observed in mice were measured every 7 days for five weeks. Tumor volumes were calculated according to the formula: length × width^2^/2. At the end of experiment, mice were sacrificed, and tumors were collected. L-OHP was purchased from Selleck Chemicals (Shanghai, China). For in vivo treatment, 10 mg/kg i.p per week was used. For experimental tumor metastatic model, mice were randomly divided into two groups (*n* = 6): USP15 sh-1# and sh-NC. USP15 sh-1# or sh-NC SGC7901-luc cells (5 × 10^6^ cells in a 100 μL volume per mouse) were injected into the tail vein of BALB/c nude mice. After five weeks, mice were sacrificed, and lungs were excised from the body. Bouin’s solution was injected from the main bronchi to fix the lung tissues.

### Immunofluorescence and confocal imaging

Immunofluorescence was implemented as previously reported. Briefly, USP15 knockdown or negative control GC cells were fixed in 4% paraformaldehyde for 20 min, cultured in blocking buffer, then incubated with primary antibodies against USP15, HDKC1, and IGF2BP3, followed by incubation with Alexa Flour donkey anti-rabbit and donkey anti-mouse antibodies (Invitrogen). The DAPI (Invitrogen) binding to DNA can be used to observe nuclear condensation. The images were obtained by laser scanning confocal microscope LSM 780 with Zen software under a 63 × oil-immersed lens (Carl Zeiss, Toronto, ON, Canada).

### Statistical analysis

All the data were expressed as the mean ± standard deviation (SD). GraphPad Prism 8.0 was used for our statistical analysis. For two-group comparisons, Student’s t-test was used. For multiple group comparisons, one-way ANOVA was used with Bonferroni post-test for comparisons between selected two groups as well as Dunnett post-test for comparisons among all other treatment groups to the corresponding control. The survival curves were drawn by Kaplan–Meier analysis, and the log-rank test was used to compare the survival differences. Differences with a value of *P* < 0.05 was regarded as statistically significant.

Further methodology details can be found in the Supplementary Information.

## Results

### USP15 is aberrantly highly expressed in GC tissues and indicative of malignant progression

**Fig. 1:**
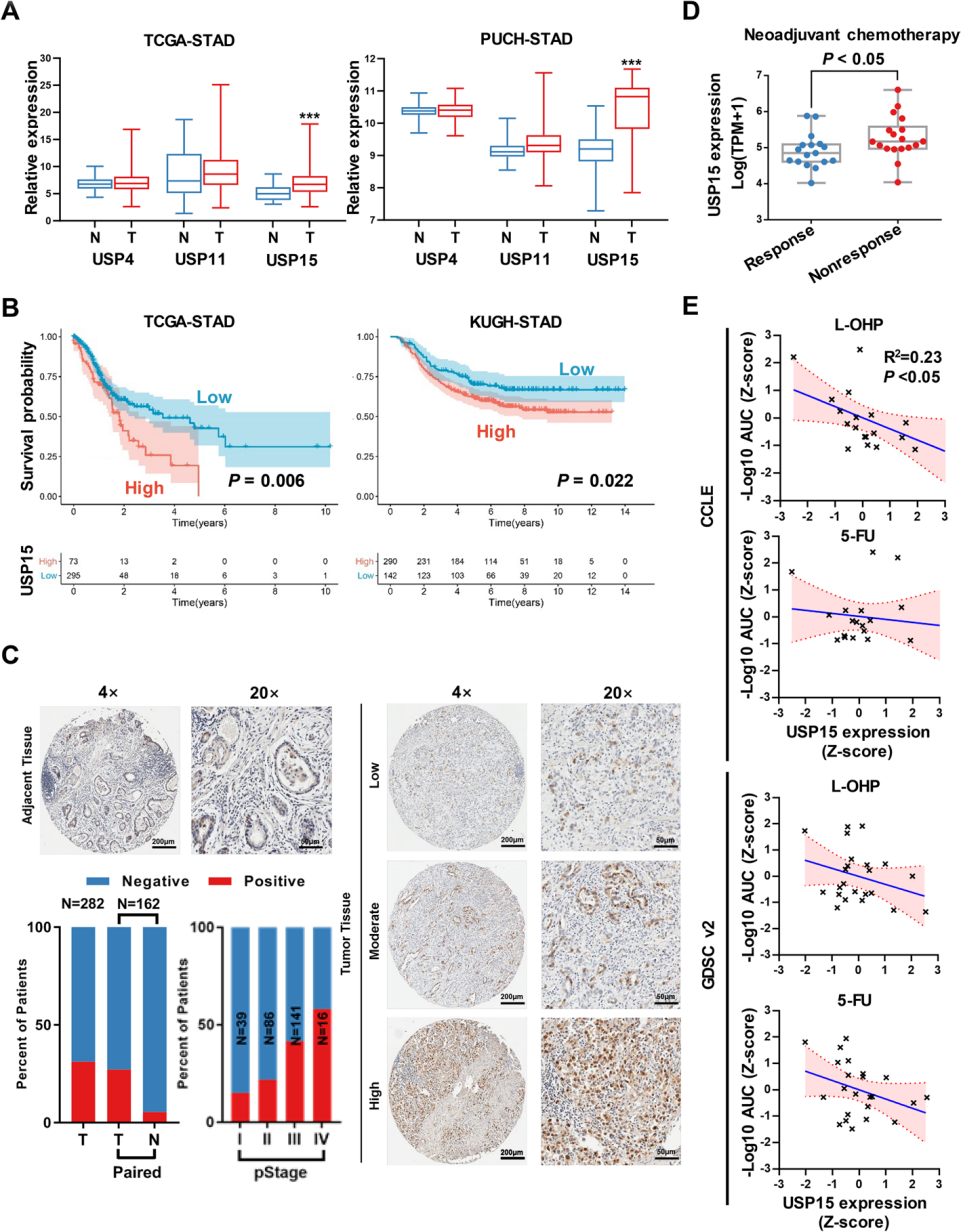
High expression of USP15 is a marker for poor prognosis and chemotherapy resistance during GC progression. **A** The expression of USP15 was analyzed in GC tissues and adjacent normal tissues from The Cancer Genome Atlas (TCGA) cohort and Peking University Cancer Hospital (PUCH) cohort. ***P < 0.001 calculated by unpaired Student’s *t*-test. **B** Kaplan–Meier plots for the overall survival rate of patients with GC in group of USP15 high or low expression level in the TCGA cohort and KUGH cohort (GEO access number: GSE26253). The *P*-values as indicated are calculated by the log-rank test. **C** Representative images of immunohistochemical (IHC) staining of USP15 in GC tissues and adjacent normal tissues provided by Biobank of Peking University Cancer Hospital. Scale bar as indicated. USP15 staining scores were further evaluated by pathologists and correlated with clinicopathological characteristics. **D** Analysis of USP15 expression in endoscopic samples with the efficacy differences in patients receiving neoadjuvant therapy (NAT). The raw data derived from NAT cohort of PUCH (https://doi.org/10.1126/sciadv.aay4211, NGDC access number: HRA000067). **E** Correlation analysis of the sensitivity of chemotherapeutic drugs and the expression level of USP15 in GC cells provided by Cancer Cell Line Encyclopedia (CCLE) database and Genomics of Drug Sensitivity in Cancer (GDSC) database. The expression levels of USP15 were normalized by the z-score method to better demonstrate. -Log_10_AUC represents the sensitivity of drug, and lower values indicate that the cells are less sensitive to the drug. The *P*-values as indicated are calculated by Pearson correlation analysis

To evaluate whether USP15 is meaningful in cancer, we first looked into public databases. The Cancer Genome Atlas (TCGA) database showed that USP15 was amplified in many types of tumors, including GC (Fig. S1A). The mRNA level of USP15 in most GC cell lines was upregulated based on the Cancer Cell Line Encyclopedia (CCLE) database (Fig. S1B). In both the TCGA-STAD and PUCH-STAD cohorts, USP15 expression levels were higher in gastric cancer (GC) tissues compared to normal tissues (Fig. 1A). In addition, patients with a high level of USP15 had poorer overall survival than those in the low expression group (Fig. 1B). As USP15 was found upregulated in GC, we confirmed its clinical significance via using immunohistochemistry. The staining of USP15 protein ranged from low to high and located in the cytoplasm (Fig. 1C), which indicated that USP15 expression was markedly increased in GC tissue sections, whereas USP15 staining was low or negative in adjacent normal tissue sections. Consistent with previous studies, the staining score of USP15 was significantly associated with tumor-node-metastasis (TNM) stage. Interestingly, the expression level of USP15 was elevated in the group of patients who were nonresponse to neoadjuvant chemotherapy (NCT) (Fig. 1D). Furthermore, both CCLE database and Genomics of Drug Sensitivity in Cancer (GDSC) database showed that USP15 expression was negative correlated with the efficacy of multiple chemotherapeutic drugs (Fig. 1E and Fig. S1C). These results suggested that abnormal expression of USP15 might be closely related to the progression of GC.

### Establishment of stable GC cell lines and evaluating the functions of USP15 in vitro

**Fig. 2:**
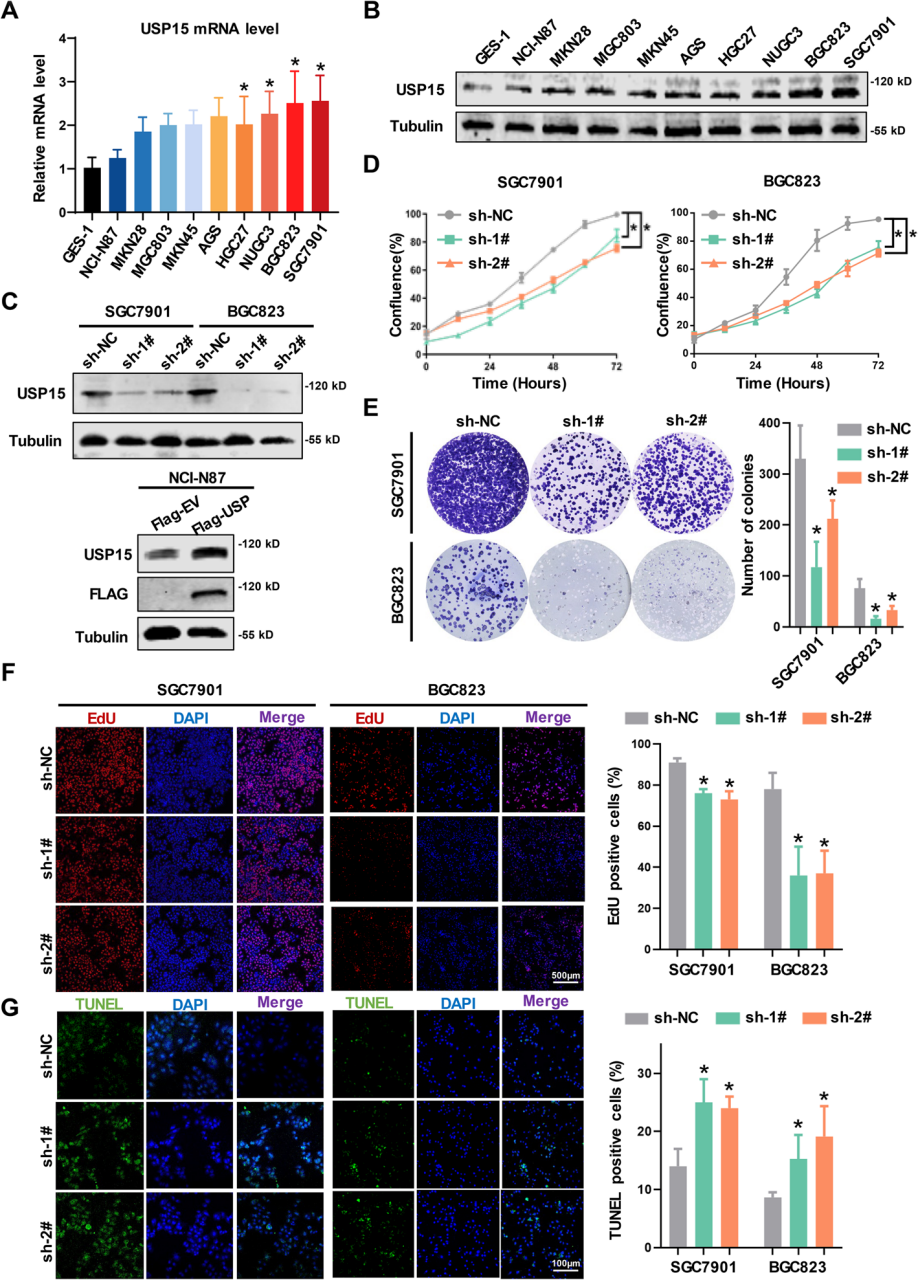
Establishing cell models with knockdown or overexpression of USP15 is performed to determine the effect of USP15 on the malignant phenotype. Relative USP15 expression levels in cell lines derived from normal gastric mucosa and primary GC by **A** qRT-PCR analysis and (**B**) western blot analysis. *P < 0.05 *vs.* GES-1. **C** Upper panel: SGC7901 and BGC823 cells were transfected with USP15 shRNAs to establish the stable knockdown cell lines. Lower panel: NCI-N87 cells were transfected with Flag-tagged USP15 ectopic expression plasmid to establish the stable overexpression cell line. The efficiencies were further confirmed by western blot analysis. β-Tubulin was used as an internal control. **D** Cell proliferation was measured by IncuCyte live cell. **E** Colony formation assay. Number of colonies was further counted. **F** The activity of DNA replication was detected by EdU staining. Scale bar = 500 μm. *P < 0.05 *vs.* sh-NC. **G** DNA strand breakage was determined by TUNEL staining. Scale bar = 100 μm. The quantitative measurements of the percentage of either EdU-stained positive cells (red) or TUNEL-stained positive cells (green) were presented in column shown in the right panel. Data are expressed as mean ± SD. *P < 0.05 *vs.* sh-NC, *n* = 12 independent experiments

To determine the cellular functions of USP15, the expression level of USP15 was detected among several cell lines. The results showed that both mRNA and protein levels of USP15 were significantly higher in SGC7901 and BGC823 cells than others (Fig. 2A and B). We synthesized two shRNA sequences to knockdown USP15 in GC cells and constructed an ectopic expression plasmid of USP15, and further established stable cell lines. The efficiency of either knockdown or overexpression of USP15 was detected by western blot (Fig. 2C). IncuCyte system for live-cell imaging and analysis was used to determine tumor cells proliferation activity, and knockdown of USP15 was consistently inhibited the cell proliferation in both SGC7901 and BGC823 cells (Fig. 2D). In addition, the colony formation ability was notably suppressed in cells with lower expression (Fig. 2E). In line with these results, the activity of DNA replication in USP15 knockdown cells was markedly decreased (Fig. 2F), which further confirmed that USP15 was involved in the regulation of aberrant proliferation signaling in GC cells. Interestingly, USP15 knockdown resulted in enhancement of DNA breakage in GC cells, as validated by the TUNEL assay (Fig. 2G). The activation of apoptotic process was confirmed via Annexin-V/PI staining and detection of death-associated proteins abundance (Fig. S2A and B).

As high expression level of USP15 was associated with metastasis in patients with primary GC, we assumed that USP15 might endow GC cells with invasive behavior. Both wound healing and invasion assays confirmed that knockdown of USP15 significantly inhibited migration and invasion (Fig. S2C and D). Besides, USP15 knockdown partially reversed the remodeling of the epithelial-mesenchymal transition (EMT), as promoting the expression of E-cadherin and reducing the expression of N-cadherin and Vimentin (Fig. S2E). Collectively, knockdown of USP15 in gastric cancer cells could lead to lower level of tumor progression.

### Knockdown of USP15 enhances the antitumor effect of chemotherapeutic drugs and distal colonization in vivo

**Fig. 3:**
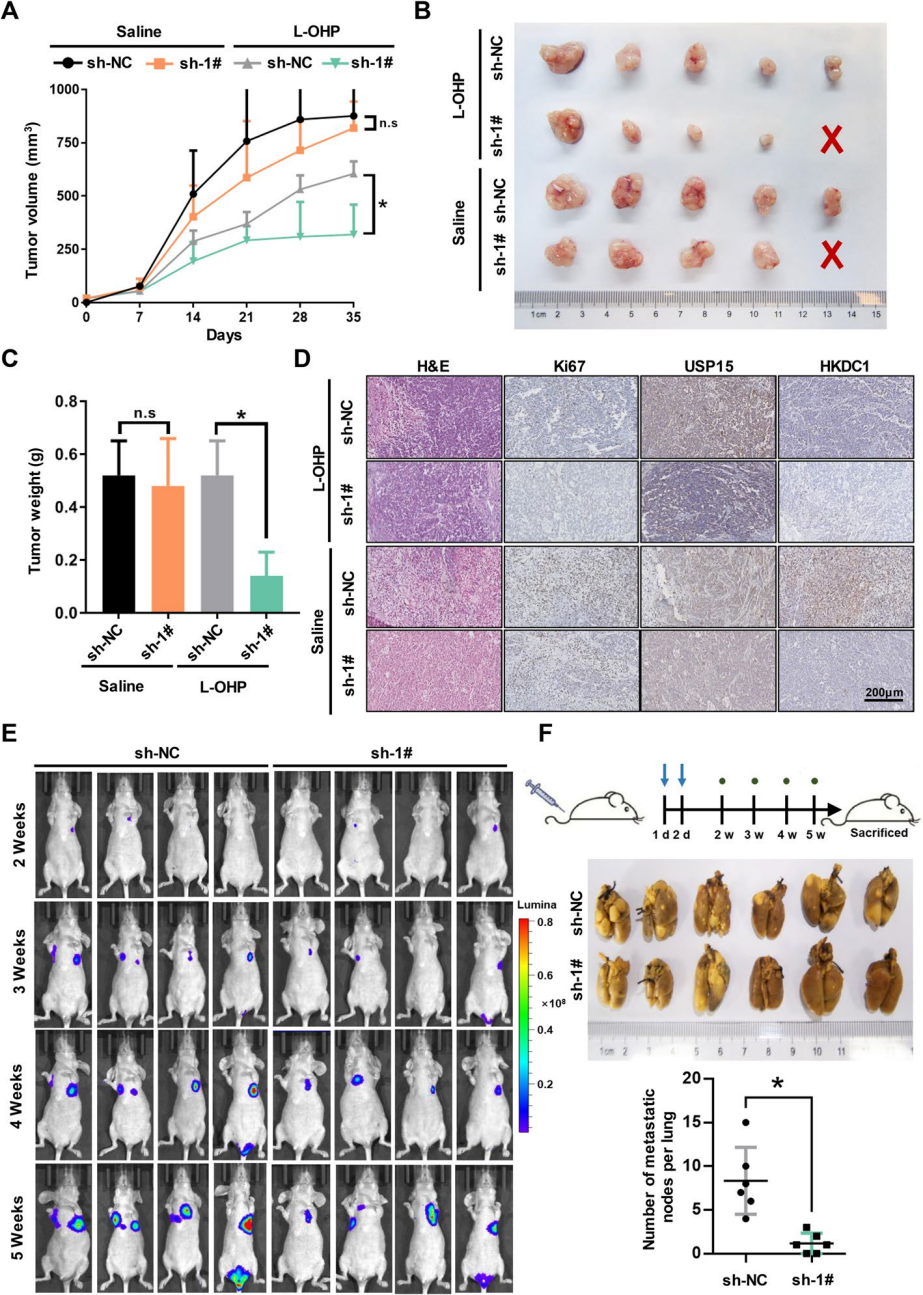
Knockdown of USP15 enhances the antitumor effect of oxaliplatin and inhibits distal colonization in xenograft models. **A** Xenograft models were established as previously described. When tumors were large enough (approximately 100 mm^3^), both sh-NC group and sh-1# group were further randomized to treatment arms. Xenograft tumor volumes measured every seven days for five weeks after treated with either oxaliplatin (L-OHP) or saline. At the end of experiment, the mice were sacrificed, and tumors were collected. **B** Representative images of xenograft tumors. **C** Tumor weight was measured. Data are expressed as mean ± SD. *P < 0.05 *vs.* sh-NC, *n* = 5 independent experiments. **D** IHC staining for Ki67, USP15, HKDC1 expression, and H&E staining of xenograft tumors. **E** The effects of USP15 knockdown on metastatic colonization through blood circulation. Either SGC7901-luc sh-NC cells or sh-1# cells were injected intravenously into mice. At each indicated time, mice were injected with D-luciferin and bioluminescence imaging was performed. Lumina intensity indicates the ability of tumor cell metastasis. **F** Representative images of Bouin-fixed lung specimens of distal colonization models and numbers of metastatic nodules in lung per mouse for each group were counted. *P < 0.05 *vs.* sh-NC, *n* = 6 independent experiments

Our previous results found that knockdown of USP15 could improve the sensitivity of chemotherapeutic drugs against GC cells, especially platinum drugs (Fig. S3A). To establish the functional importance of USP15 on the progression of GC, nude mice were subcutaneously injected with SGC7901 cells (sh-NC, sh-1#), respectively. After 1 week, xenograft tumor models could be observed growing by eyes nearly at the same time. We further divided the nude mice into four groups and treated with Oxaliplatin (L-OHP, 5 mg/Kg, i.p.) and saline respectively, twice per week, lasting for 4 weeks. Although inhibition of USP15 expression did not effectively prevent tumor growth, USP15 knockdown indeed enhanced the antitumor effect of L-OHP, which was similar with the results of in vitro (Fig. 3A-C). Moreover, the expression level of USP15 remained low in xenograft tumor tissues of the knockdown group and was consistent with the trend of proliferation markers expression (Fig. 3D and Fig. S3C). Next, we evaluated the effect of USP15 knockdown on tumor metastatic colonization in nude mice. As usual, SGC7901-luc cells stably transfected with either sh-NC or sh-1# were injected into nude mice via the tail vein. Bioluminescent imaging was performed at day 14, 21, 28, 35 and only slight distant metastases were found in mice of the USP15 knockdown group (Fig. 3E and F). These data indicated that USP15 aggressively promoted malignant progression and is expected to be a therapeutic target for GC.

### USP15 plays an important role in glycolytic remodeling in GC progression

**Fig. 4:**
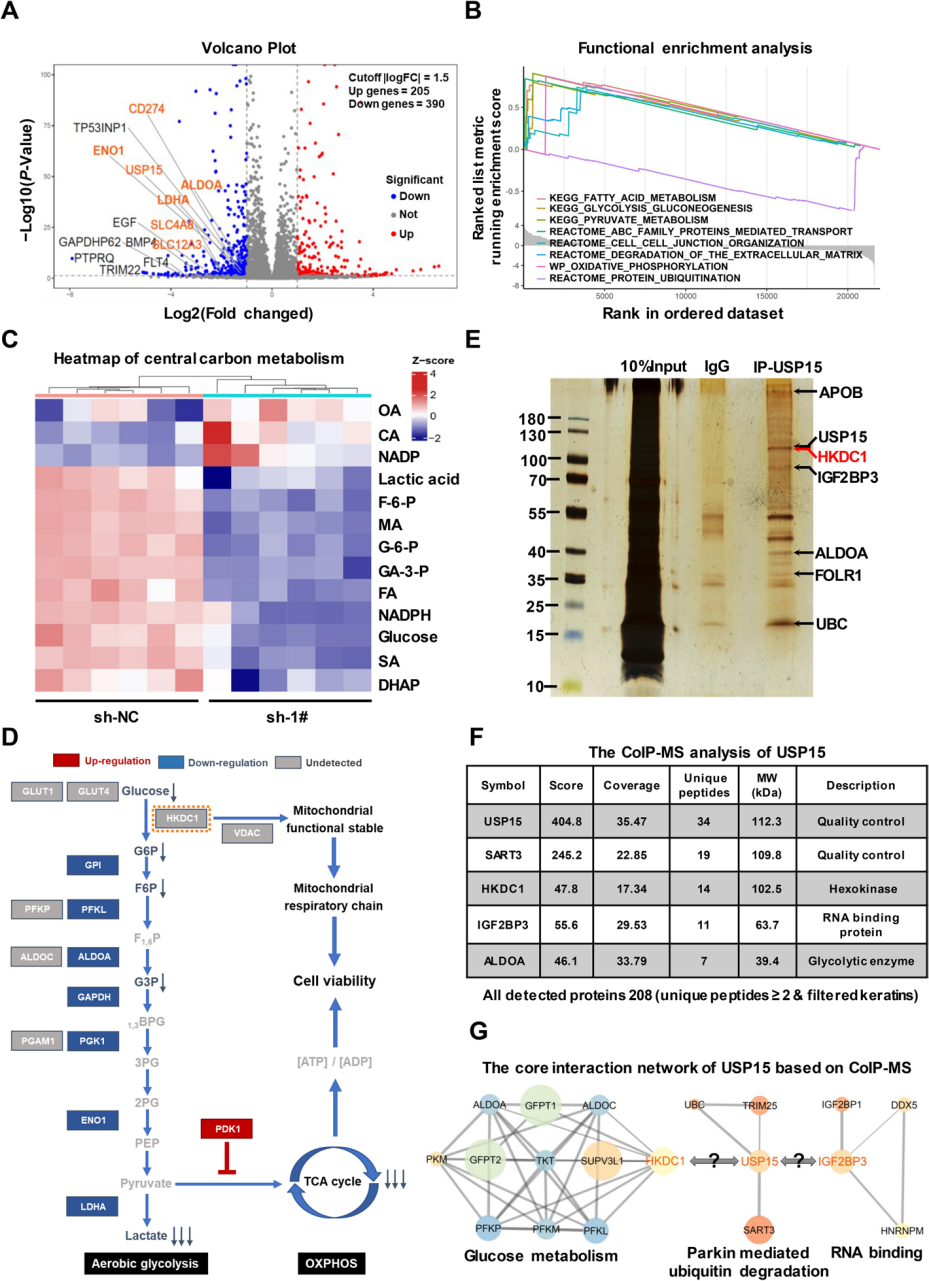
Prediction of potential biological functions and targets of USP15. **A** RNA sequencing analysis was performed to detect differentially expressed genes in GC cells following USP15 knockdown, and volcano plot was displayed with tumor driver genes (e.g., EGF, CD274) and metabolic key enzymes (e.g., ALDOA, LDHA) as indicated. **B** Functional enrichment analysis based on GSEA was performed. Knockdown of USP15 significantly affect the regulation of multiple glucose metabolic pathways, including glycolysis, oxidative phosphorylation (OXPHOS). **C** Heatmap of the average order of magnitude of central carbon metabolites. **D** Simplified schematic overview of central carbon metabolism in *Homo sapiens*, with heatmap of the log2 fold change of average metabolite and mRNA levels in sh-1# cells versus sh-NC cells. Two-tailed T-test, adjusted *P*-value (False Discovery Rate (FDR)) = 0.05. Metabolites and related genes colored light grey were not detected or unchanged. Co-enzymes and substrates are not included. **E** Co-immunoprecipitation experiments were performed using SGC7901 cell lysates with anti-USP15 antibody, or with IgG as negative control. The proteins were resolved on SDS-PAGE and stained with silver staining. **F** The specific peptides in the USP15 complex were resolved using MS, and the bands of potential targets supported by MS assay were indicated by arrows shown in the (**E**). **G** Analysis of protein–protein interaction network of USP15 based on CoIP-MS

To predict the potential downstream regulatory mechanisms of USP15 signaling axis, we performed a combined multi-omics analysis through transcriptome profiling, targeted metabolome profiling, and interacting protein identification profiling. The transcriptome sequencing identified a set of differentially expressed genes after USP15 knockdown and presented these genes via volcano plot and heatmap (Fig. 4A and Fig. S4A) The result of functional enrichment analysis showed high confidence of genes enriched in the function of ubiquitination, degradation of the extracellular matrix, pyruvate metabolism, and glycolysis (Fig. 4B and S4B). Notably, knockdown of USP15 significantly reduced the production of glycolytic process in GC cells, including lactic acid, fructose-6-phosphate (F-6-P), and glucose-6-phosphate (G-6-P), which was consistent with the canonical molecules of these pathways significantly modulated (Fig. 4C and D and Fig. S4C). Besides, low expression of USP15 has a lower metabolism signature score in TCGA-STAD cohort, especially for glycolysis and OXPHOS activity (Fig. S4D). Identification of USP15-interacting proteins was performed using CoIP-MS based proteomics, and silver staining was conducted to distinguish the differential protein bands in the resultant immunoprecipitants (Fig. 4E). These immunoprecipitants was next analyzed by MS, and the unique proteins captured by USP15 antibody were compared to previous studies that reported USP15 interaction (Fig. 4F and Table S4). In line with transcriptomics and metabolomics results, CoIP-MS analysis showed that some core regulators in the glycolytic pathway bind to USP15, including HKDC1 and IGF2BP3 (Fig. 4G and Fig. S4E), suggesting that USP15 might be directly involved in the ubiquitination modification of these proteins.

### Knockdown of USP15 suppresses glycolytic activity and impairs mitochondrial functions

**Fig. 5:**
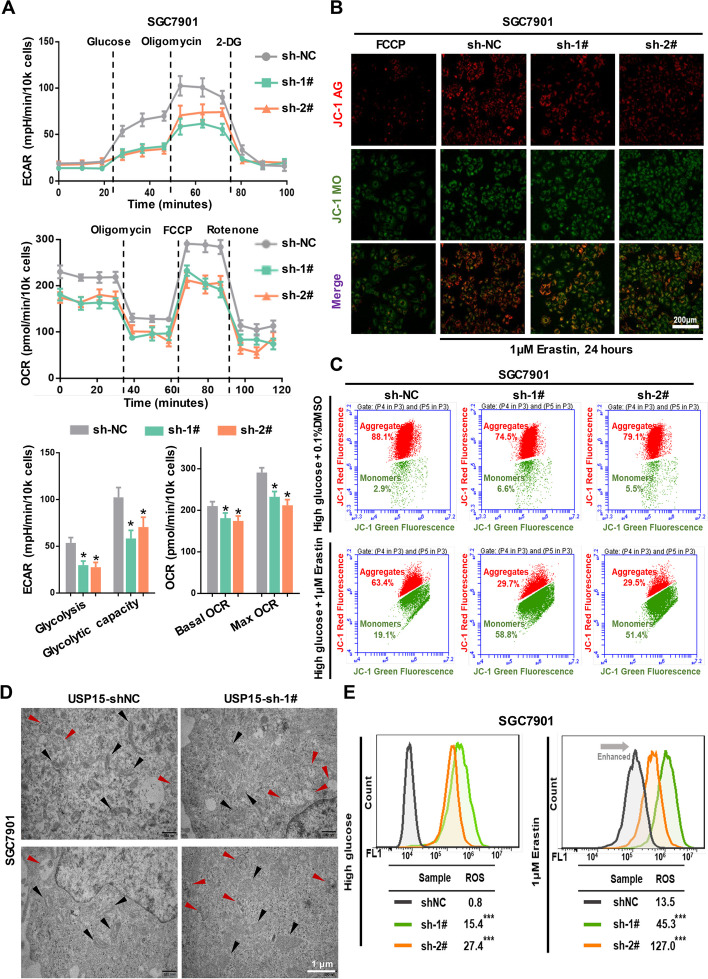
Knockdown of USP15 inhibits glycolytic activity and causes both functional and structural damage to mitochondria. **A** Both ECAR and OCR of stable USP15-knockdown or negative control SGC7901 cells were measured. For ECAR, the Seahorse automatically filled each well with 10 mmol/L glucose, 1 µmol/L oligomycin, and 50 mmol/L 2-DG successively. Basal glycolysis was determined after addition of glucose, and glycolytic capacity was calculated after addition of oligomycin. For OCR, 1 µmol/L oligomycin, 1 µmol/L FCCP, and 0.5 µmol/L rotenone were automatically injected successively. Basal OCR was determined prior to addition of oligomycin, and maximal respiratory capacity was determined by subtracting nonmitochondrial OCR, calculated after rotenone injection to maximal OCR upon FCCP uncoupling and maximal electron transport. Data are expressed as mean ± SD. *P < 0.05 *vs.* sh-NC, *n* = 12 independent experiments. **B** Mitochondrial membrane potential of stable USP15-knockdown or negative control SGC7901 cells was detected by JC-1 probe. The red fluorescence represents the mitochondrial aggregate JC-1, and the green fluorescence indicates the monomeric JC-1. Scale bar = 200 μm. **C** Changes of mitochondrial membrane potential were further measured quantitatively by FACS analysis. Erastin was used to trigger the mitochondrial stress in GC cells. **D** Representative transmission electron microscope (TEM) images of the morphological and subcellular structure of different groups. Red arrows represent mitochondria with abnormal structure, and black arrows represent mitophagy. Scale bar = 1 μm. **E** Intracellular ROS level was assayed by FACS analysis using DCFH-DA fluorescent probe. Data are expressed as mean ± SD. ***P < 0.001 *vs.* sh-NC, *n *= 10 independent experiments

The above indicated that USP15 may be an important component for the regulation of glycolytic activity in GC cells, thereby we measured the effects of inhibition of USP15 expression on glycolysis and mitochondrial OXPHOS processes. USP15 knockdown not only resulted in blocking of glycolysis and glycolytic capacity, but also decreased the levels of both basal O_2_ consumption rate (OCR) and max OCR (Fig. 5A), indicating compromised aerobic glycolysis and aerobic oxidation. As the integrity of mitochondria is important for maintaining the OXPHOS process as well as tumor cell viability, we analyzed the functional and structural integrity of mitochondria in GC cells after USP15 knockdown. The JC-1 probe was used to detect the mitochondrial membrane potential to indicate the functionality of the mitochondria. The results showed that USP15 knockdown induced an abnormal reduction in the mitochondrial membrane potential, as the fluorescent pattern changed from a punctate red fluorescence to a diffuse green fluorescence (Fig. 5B and Fig. S5A). Besides, the number of cells with abnormal mitochondria increased significantly after knockdown of USP15 (Fig. 5C and Fig. S5B). Importantly, transmission electron microscope imaging clearly observed the abnormalities of mitochondrial structure in GC cells caused by USP15 knockdown (Fig. 5D and Fig. S5C). Elevated intracellular ROS levels and aberrant expression of mitochondria-related proteins further indicated the mitochondrial damage in GC cells following USP15 knockdown (Fig. 5E and Fig. S6).

### The glycolytic regulators HKDC1 and IGF2BP3 are the core interacting targets of USP15

**Fig. 6:**
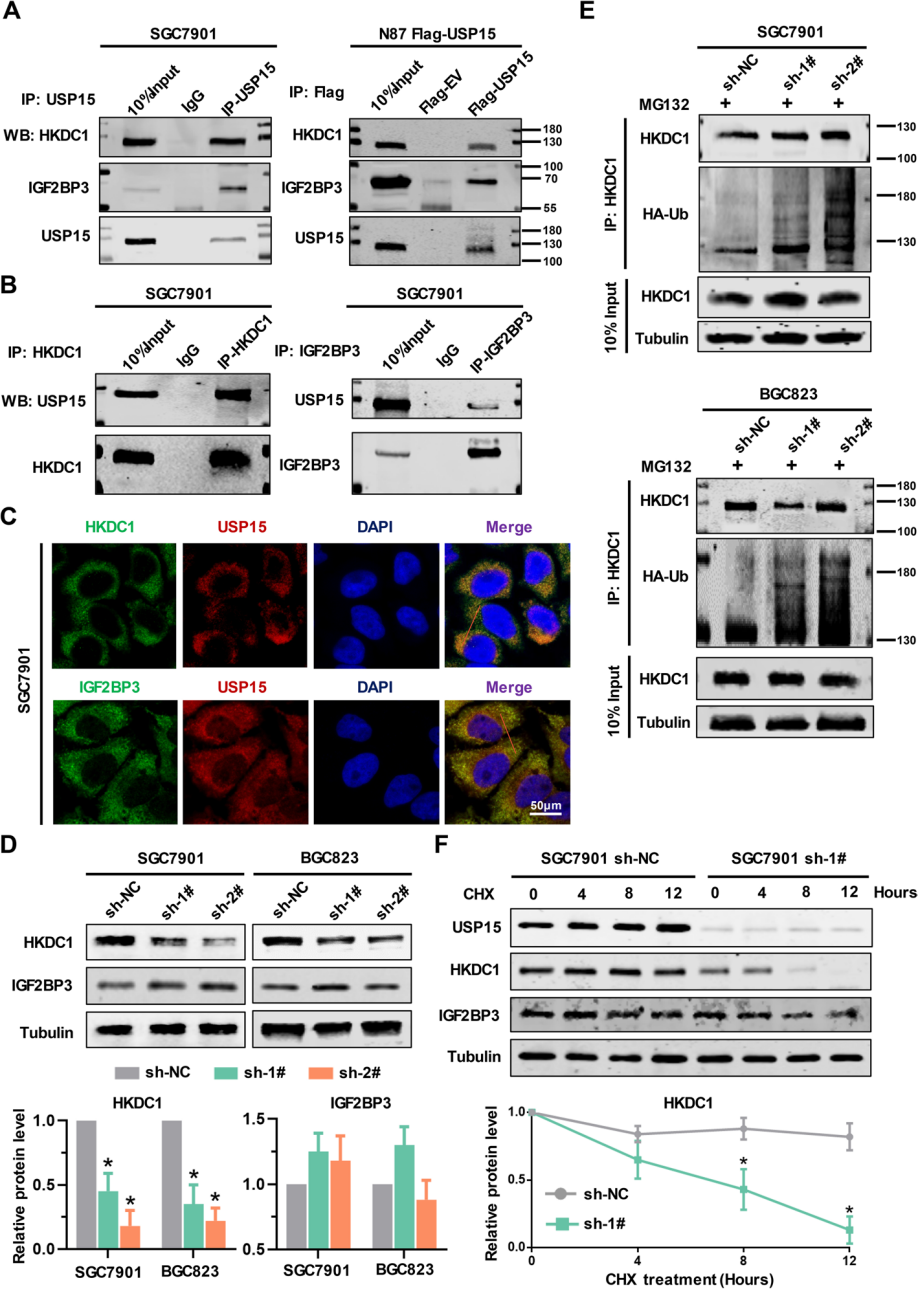
USP15 interacts with both HKDC1 and IGF2BP3. **A** Left panel: Cell lysates were extracted from SGC7901 cells. Immunoprecipitated with anti-USP15 antibody, or with IgG as negative control. Right panel: Cell lysates were extracted from N87 Flag-USP15 cells or Flag tagged empty vector (Flag-EV) transfected cells. Immunoprecipitated with anti-Flag. HKDC1, IGF2PB3, and USP15 were further detected by western blot analysis. **B** Immunoprecipitation experiments were performed using SGC7901 cell lysates with anti-HKDC1 antibody (left panel) or anti-IGF2BP3 antibody (right panel), IgG was used as negative control. HKDC1, IGF2PB3, and USP15 were further detected by western blot analysis. **C** Colocalization analysis by dual-color confocal imaging. Scale bar = 50 μm. **D** Western blot analysis was performed to determine the expression levels of HKDC1 and IGF2BP3 following USP15 knockdown. Data are expressed as mean ± SD. *P < 0.05 *vs.* sh-NC, *n* = 6 independent experiments. **E** GC cells were co-transfected with HA-Ub and USP15 shRNAs. MG132, a proteasome inhibitor, was used to treated with among USP15 sh-1#, sh-2#, and sh-NC cells. After 4 h treated with 5 μM MG132, immunoprecipitation experiments were performed using these cell lysates with anti-HKDC1 antibody. The ubiquitination level of HKDC1 was detected by western blot using anti-HA antibody. **F** Cycloheximide (CHX, 50 μM), a protein synthesis inhibitor, was used to treated with USP15 sh-1# and sh-NC cells. The accumulation levels of both HKDC1 and IGF2BP3 were evaluated by western blot analysis. β-Tubulin was used as an internal control. Data are expressed as mean ± SD. *P < 0.05 *vs.* sh-NC, *n* = 6 independent experiments

Combined with the multi-omics results, we hypothesized that hexokinase HKDC1 and IGF-binding protein IGF2BP3 are the key targets of USP15 downstream. The CoIP experiments were performed to verify the interaction of USP15 with HKDC1 or IGF2BP3. The results confirmed that both HKDC1 and IGF2BP3 interacted with USP15 directly (Fig. 6A and Fig. 6B), and those proteins colocalize at the subcellular level (Fig. 6C and Fig. S7). Given that USP15 is a regulator on the glycolysis, we wonder whether USP15 would be a deubiquitinase for both HKDC1 and IGF2BP3. We further determined the role of USP15 in the expression of HKDC1 and IGF2BP3. Interestingly, knockdown of USP15 reduced the protein level of HKDC1 without affecting IGF2BP3 (Fig. 6D). After blocking the proteasome activity by treating cells with MG132, the decreased protein level of HKDC1 induced by USP15 knockdown was rescued, indicating that USP15 is responsible for the stability of HKDC1 protein. Importantly, USP15 knockdown notably increased the accumulation of ubiquitinated HKDC1 (Fig. 6E). Considering the synthesis pathway, cycloheximide (CHX), a protein synthesis inhibitor, was used to detect the half-life of protein. Western blot analysis showed that USP15 knockdown decreased the half-life of HKDC1 rather than IGF2BP3 (Fig. 6F).

To further evaluate whether the enzymatic activity of USP15 is essential for the deubiquitination of HKDC1, we transfected USP15 knockdown cell lines with expression vectors for USP15 variants, USP15-298A (loss of enzyme activity) and USP15-812A (loss of activity towards polyubiquitin). Ectopic expression of USP15-298A mutant resulted in decreased protein level of HKDC1 (Fig. S8A). The up-regulated level of ubiquitination of HKDC1 caused by repression of USP15 was reversed by USP15 wild-type, but not by the 298A mutant, whereas USP-812A mutant showed differently (Fig. S8B). As expected, the attenuation of glycolytic activity in GC cells was rescued by overexpression of USP15 wild type (USP15-WT), rather than 298A mutant (Fig. S9A). Importantly, overexpression of USP15 mutants deficient in enzymatic activity in USP15 knockdown cells failed to enhance the activities of both proliferation and DNA replication in GC cells (Fig. S9B and C). In addition, only USP15-WT was able to inhibit apoptosis and maintain mitochondrial stability in GC cells (Fig. S10A and B). All these results suggest that deubiquitination of HKDC1 requires the enzymatically active USP15, which regulates glycolytic activity.

### HKDC1 is a key target of USP15 to regulate glycolytic activity

**Fig. 7:**
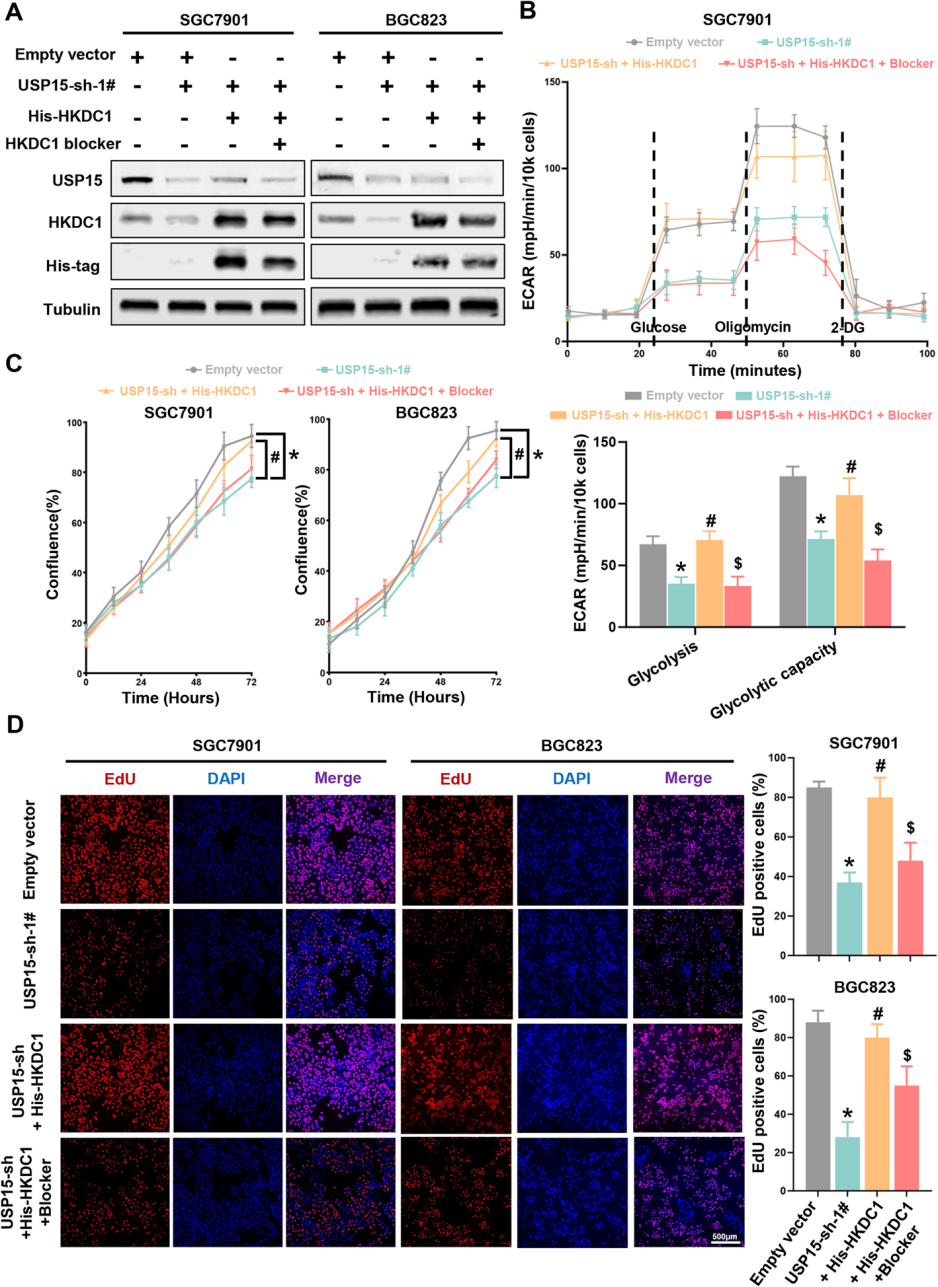
USP15 regulated glycolysis through HKDC1. **A** Western blot analysis was performed to determine the expression levels of USP15, HKDC1 and His-tag after treating with HKDC1 blocker. β-Tubulin was used as an internal control. **B** ECAR of USP15-sh-1#, USP15-sh-His-HKDC1, USP15-sh-His-HKDC1 + blocker and empty vector cells were measured. Data are expressed as mean ± SD. *P < 0.05 *vs.* empty vector, #*P* < 0.05 *vs.* USP15-sh-1#, $*P* < 0.05 *vs.* USP15-sh + His-HKDC1, *n* = 8 independent experiments. **C** Cell proliferation was measured by IncuCyte system. **D** The activity of DNA replication was detected by EdU staining. Scale bar = 500 μm. Data are expressed as mean ± SD. *P < 0.05 *vs.* empty vector, #*P* < 0.05 *vs.* USP15-sh-1#, $*P* < 0.05 *vs.* USP15-sh-His-HKDC1, *n* = 12 independent experiments

To further determined whether HKDC1 is essential for the regulation of glycolytic activity by USP15 in GC, we used HKDC1 blocker or siRNA to interfere with HKDC1 function or expression. Overexpression of HKDC1 enhanced glycolytic activity and can be reversed by HKDC1 blocker in GC cells (Fig. 7A and B and Fig. S11). Moreover, HKDC1 blocker decreased the activates of both proliferation and DNA replication (Fig. 7C and D), which were enhanced by overexpression of HKDC1. Similarly, HKDC1 silencing in NCI-N87 cells reversed the enhancement of glycolytic activity induced by USP15 up-regulation (Fig. S13A and B). Besides, in line with the results of HKDC1 blocker, the cell viability was also suppressed by HKDC1 silencing (Fig. S13C and D). These data confirmed that HKDC1 is required for USP15 regulated glycolytic activity in GC.

## Discussion

Comparatively less is presently known on the counteracting mechanisms that rely on deubiquitinases (DUBs). These enzymes are commonly aberrantly expressed in various cancers and promote malignant progression [15]. It has been reported that DUBs can directly regulate the function of oncoproteins. For example, USP7 interacts with estrogen receptor alpha (ERα) and mediates the stability of PHF8, an important histone demethylase, to promote breast carcinogenesis [16]. USP14 controls epidermal growth factor receptor (EGFR) fate in lung cancer by deubiquitinating the endocytic adaptor Eps15 [17]. An oncogenic activity of USP15 was previously suggested by its function to deubiquitylate and stabilize the TGFBR and MDM2 [18, 19]. Analysis of a pan-cancer project showed that USP15 was amplified in about 4% of different type of cancers, including GC [20]. However, mechanism underlying the oncogenic functions of USP15 remains unclear. Recently, another team and we have successively reported that USP15 is abnormally upregulated in GC [9, 10, 21]. The results have shown that USP15 acted as an oncogene, thereby promoting invasion metastasis, and progression of GC. Besides, we further identified the mechanism by which USP15 is aberrantly expressed through the miR-26a signaling axis. In this research, we provide the first landscape of the key functionals of USP15 downstream with the core interaction targets through multi-omics analysis. The results confirm that USP15 is an important regulator in driving metabolic reprogramming, affecting glycolytic activity and mitochondrial functions in GC cells.

Hexokinase (HK) family is a group of key rate-limiting enzymes in the glycolysis, and some of them also maintain structural as well as functional stability of mitochondria [22]. HKDC1 is a novel HK isoform that is involved in glucose utilization and mitochondrial voltage-dependent anion channel (mVDAC)-mediated counteracting metabolic stress processes [23]. IGF2BP3 is an RNA-binding protein that belongs to the IGF2BP family, which plays oncogenic role in multiple cancers [24]. Notably, both HKDC1 and IGF2BP3 are essential for the regulation of glycolytic activity, and abnormally high expression of them has been reported in GC, which is consistent to current multidrug chemotherapies with a poor prognosis [25–27]. It has been reported that HKDC1 can be degraded by ubiquitination system [28], but deubiquitinases, which can protect ubiquitinated proteins from degradation, were less to know. For the first time, we report that HKDC1 is potential substrates for USP15. However, we only demonstrate that the expression level of HKDC1 is tightly regulated by ubiquitination modification that can inhibit the degradation of HKDC1, at least through USP15 deubiquitination. According to our current results, IGF2BP3 may not be a substrate for USP15, but is most likely a USP15 binding protein that plays a guiding role in substrate recognition, as it can bind multiple RNA sequences. Although our current study brings forward previously unknown players HKDC1 and IGF2BP3 in GC progression, it also raises many questions that need to be addressed in future studies.

The findings presented here also bear important therapeutic implications. Considering that the mechanisms of drug action for HKDC1 and IGF2BP3 differ significantly, the potential of drugs targeting both is likely to be more efficient than targeting independent mechanisms in the tumor cell. DUBs are a newly emerging class of drug targets, and potent and selective DUBs inhibitors are being developed [29]. Following the potential substrates of USP15 being explored, our findings highlight that USP15 could be considered as a novel target for therapeutic intervention in GC. Knockdown of USP15 could reverse malignant progression both in vitro and in vivo. Besides, we found that the expression level of USP15 was negatively correlated with chemotherapeutic drug sensitivity, especially for platinum-based drugs. This may be related to the functions of USP15 downstream, since glycolytic capacity is thought to be a factor affecting drug sensitivity while USP15 knockdown impaired it. In other words, the expression level of USP15 might be a predictor of chemotherapy efficacy in patients.

Current studies deeply explored the clinical significance and molecular functions of USP15 in GC. However, there are some obvious limitations in our research. The effect on glucose metabolism is remarkably specific to USP15, but whether the closely related USP4 and USP11 have the same effect has not been investigated. Although we identified a set of USP15 interacting proteins and verified the binding of both HKDC1 and IGFPB3 to USP15, the existence of other key interacting proteins needs further analysis. The identification of either substrates or regulators of USP15 also leaves much to explore.

## Conclusions

Collectively, our research demonstrated that USP15 was upregulated in GC cells and tissues, and high expression of USP15 was associated with poor prognosis and chemoresistance in patients with GC. Notably, our results supported USP15 as a key regulator of glucose metabolism, which has not been reported in previous studies. In addition, we identified a set of USP15 interacting proteins, including HKDC1 and IGF2BP3. Importantly, inhibition of USP15 expression reduced glycolytic activity and block the glucose metabolic process, leading to mitochondrial damage, which might be regulated through HKDC1. Interfering with USP15 expression reversed tumor progression and distal colonization in vivo. Further experiment confirmed that the enzymatic activity of USP15 is essential for the deubiquitination of HKDC1. Together with the findings presented above, we suggest that USP15 inhibitors, if developed, could be specific and effective in promoting chemotherapy through glucose metabolism remodeling with limited toxicity.

### Supplementary Information

Supplementary Material 1

## Data Availability

All data supports the findings of this study are included in this published article and available in the Supporting Information Material of this article.

## Abbreviations

USP15: Ubiquitin-specific protease 15
GC: Gastric cancer
NAT: Neoadjuvant therapy
USPs: Ubiquitin-specific proteases
OXPHOS: Oxidative phosphorylation
HKDC1: Hexokinase domain containing 1
IGF2BP3: Insulin like growth factor 2 mRNA binding protein 3
TNM: Tumor-node-metastasis
DMEM: Dulbecco’s modified Eagle’s medium
PCR: Polymerase chain reaction
SD: Standard deviation
TCGA: The Cancer Genome Atlas
CCLE: Cancer Cell Line Encyclopedia
NCT: Neoadjuvant chemotherapy
GDSC: Genomics of Drug Sensitivity in Cancer
EMT: Epithelial-mesenchymal transition
L-OHP: Oxaliplatin
F-6-P: Fructose-6-phosphate
G-6-P: Glucose-6-phosphate
OCR: O_2_ consumption rate
CHX: Cycloheximide
EGFR: Epidermal growth factor receptor
FDR: False Discovery Rate
Flag-EV: Flag tagged empty vector
HK: Hexokinase
mVDAC: Mitochondrial voltage-dependent anion channel
NC: Nitrocellulose
CoIP: Co-immunoprecipitation
CT: Cycle threshold
PI: Propidium iodide
TEM: Transmission electron microscopy
ECAR: Extracellular acidification rate

## References

[1] Bray F, Jemal A, Grey N, Ferlay J, Forman D. Global cancer transitions according to the Human Development Index (2008–2030): a population-based study. Lancet Oncol. 2012;13(8):790–801. 22658655 10.1016/S1470-2045(12)70211-5

[2] Matsuda T, Gatellier L. Projection of the number of new stomach cancer cases in the world. Jpn J Clin Oncol. 2023;53(8):741–2. 37477363 10.1093/jjco/hyad084

[3] Li S, Shan F, Zhang X, Li Y, Sun Y, Tang L, et al. Chinese quality control indices for standardized diagnosis and treatment of gastric cancer (2022 edition). Chin J Cancer Res. 2022;34(6):623–32. 36714343 10.21147/j.issn.1000-9604.2022.06.10 PMC9829494

[4] Song JH, Han SU. Perspectives of laparoscopic surgery for gastric cancer. Chin J Cancer Res. 2022;34(5):533–8. 36398120 10.21147/j.issn.1000-9604.2022.05.12 PMC9646463

[5] Sun T, Liu Z, Yang Q. The role of ubiquitination and deubiquitination in cancer metabolism. Mol Cancer. 2020;19(1):146. 33004065 10.1186/s12943-020-01262-x PMC7529510

[6] Young MJ, Hsu KC, Lin TE, Chang WC, Hung JJ. The role of ubiquitin-specific peptidases in cancer progression. J Biomed Sci. 2019;26(1):42. 31133011 10.1186/s12929-019-0522-0 PMC6537419

[7] Ma C, Tian Z, Wang D, Gao W, Qian L, Zang Y, et al. Ubiquitin-specific protease 35 promotes gastric cancer metastasis by increasing the stability of Snail1. Int J Biol Sci. 2024;20(3):953–67. 38250150 10.7150/ijbs.87176 PMC10797686

[8] Li YC, Cai SW, Shu YB, Chen MW, Shi Z. USP15 in cancer and other diseases: from diverse functionsto therapeutic targets. Biomedicines. 2022;10(2):474. 35203682 10.3390/biomedicines10020474 PMC8962386

[9] Cui Z, Sun H, Gao Z, Li C, Xiao T, Bian Y, et al. TRIM21/USP15 balances ACSL4 stability and the imatinib resistance of gastrointestinal stromal tumors. Br J Cancer. 2024;130(4):526–41. 38182686 10.1038/s41416-023-02562-x PMC10876985

[10] Huangfu L, Fan B, Wang G, Gan X, Tian S, He Q, et al. Novel prognostic marker LINC00205 promotes tumorigenesis and metastasis by competitively suppressing miRNA-26a in gastric cancer. Cell Death Discov. 2022;8(1):5. 35013132 10.1038/s41420-021-00802-8 PMC8748761

[11] Yuan LW, Yamashita H, Seto Y. Glucose metabolism in gastric cancer: the cutting-edge. World J Gastroenterol. 2016;22(6):2046–59. 26877609 10.3748/wjg.v22.i6.2046 PMC4726677

[12] Liu Y, Zhang Z, Wang J, Chen C, Tang X, Zhu J, et al. Metabolic reprogramming results in abnormal glycolysis in gastric cancer: a review. Onco Targets Ther. 2019;12:1195–204. 30863087 10.2147/OTT.S189687 PMC6389007

[13] Xie Y, Wang M, Xia M, Guo Y, Zu X, Zhong J. Ubiquitination regulation of aerobic glycolysis in cancer. Life Sci. 2022;292: 120322. 35031261 10.1016/j.lfs.2022.120322

[14] Ma L, Zong X. Metabolic symbiosis in chemoresistance: refocusing the role of aerobic glycolysis. Front Oncol. 2020;10: 5. 32038983 10.3389/fonc.2020.00005 PMC6992567

[15] Deng L, Meng T, Chen L, Wei W, Wang P. The role of ubiquitination in tumorigenesis and targeted drug discovery. Signal Transduct Target Ther. 2020;5(1):11. 32296023 10.1038/s41392-020-0107-0 PMC7048745

[16] Xia X, Liao Y, Huang C, Liu Y, He J, Shao Z, et al. Deubiquitination and stabilization of estrogen receptor alpha by ubiquitin-specific protease 7 promotes breast tumorigenesis. Cancer Lett. 2019;465:118–28. 31518603 10.1016/j.canlet.2019.09.003

[17] Wang S, Wang T, Yang Q, Cheng S, Liu F, Yang G, et al. Proteasomal deubiquitylase activity enhances cell surface recycling of the epidermal growth factor receptor in non-small cell lung cancer. Cell Oncol (Dordr). 2022;45(5):951–65. 36129611 10.1007/s13402-022-00699-0 PMC12978065

[18] Zou Q, Jin J, Hu H, Li HS, Romano S, Xiao Y, et al. USP15 stabilizes MDM2 to mediate cancer-cell survival and inhibit antitumor T cell responses. Nat Immunol. 2014;15(6):562–70. 24777531 10.1038/ni.2885 PMC4032322

[19] Eichhorn PJ, Rodon L, Gonzalez-Junca A, Dirac A, Gili M, Martinez-Saez E, et al. USP15 stabilizes TGF-beta receptor I and promotes oncogenesis through the activation of TGF-beta signaling in glioblastoma. Nat Med. 2012;18(3):429–35. 22344298 10.1038/nm.2619

[20] Das T, Song EJ, Kim EE. The multifaceted roles of USP15 in signal transduction. Int J Mol Sci. 2021;22(9):4728. 33946990 10.3390/ijms22094728 PMC8125482

[21] Zhong M, Zhou L, Fang Z, Yao YY, Zou JP, Xiong JP, et al. Ubiquitin-specific protease 15 contributes to gastric cancer progression by regulating the Wnt/beta-catenin signaling pathway. World J Gastroenterol. 2021;27(26):4221–35. 34326621 10.3748/wjg.v27.i26.4221 PMC8311539

[22] Xu S, Herschman HR. A tumor agnostic therapeutic strategy for hexokinase 1-null/hexokinase 2-positive cancers. Cancer Res. 2019;79(23):5907–14. 31434645 10.1158/0008-5472.CAN-19-1789 PMC12139393

[23] Zapater JL, Lednovich KR, Khan MW, Pusec CM, Layden BT. Hexokinase domain-containing protein-1 in metabolic diseases and beyond. Trends Endocrinol Metab. 2022;33(1):72–84. 34782236 10.1016/j.tem.2021.10.006 PMC8678314

[24] Mancarella C, Scotlandi K. IGF2BP3 from physiology to cancer: novel discoveries, unsolved issues, and future perspectives. Front Cell Dev Biol. 2019;7:363. 32010687 10.3389/fcell.2019.00363 PMC6974587

[25] Clemente-Suarez VJ, Martin-Rodriguez A, Redondo-Florez L, Ruisoto P, Navarro-Jimenez E, Ramos-Campo DJ, et al. Metabolic health, mitochondrial fitness, physical activity, and cancer. Cancers (Basel). 2023;15(3):814. 36765772 10.3390/cancers15030814 PMC9913323

[26] Zhao P, Yuan F, Xu L, Jin Z, Liu Y, Su J, et al. HKDC1 reprograms lipid metabolism to enhance gastric cancer metastasis and cisplatin resistance via forming a ribonucleoprotein complex. Cancer Lett. 2023;569: 216305. 37423558 10.1016/j.canlet.2023.216305

[27] Wang MQ, Chen YR, Xu HW, Zhan JR, Suo DQ, Wang JJ, et al. HKDC1 upregulation promotes glycolysis and disease progression, and confers chemoresistance onto gastric cancer. Cancer Sci. 2023;114(4):1365–77. 36519789 10.1111/cas.15692 PMC10067396

[28] Senft D, Qi J, Ronai ZA. Ubiquitin ligases in oncogenic transformation and cancer therapy. Nat Rev Cancer. 2018;18(2):69–88. 29242641 10.1038/nrc.2017.105 PMC6054770

[29] Nalepa G, Rolfe M, Harper JW. Drug discovery in the ubiquitin-proteasome system. Nat Rev Drug Discov. 2006;5(7):596–613. 16816840 10.1038/nrd2056

