# Supplementary material for: The deubiquitinase USP15 drives malignant progression of gastric cancer through glucose metabolism remodeling
Source: J Exp Clin Cancer Res. 2024 Aug 20;43:235. doi: 10.1186/s13046-024-03152-2 (PMC11334570; doi:10.1186/s13046-024-03152-2)
Supplement: Supplementary file 1 — Supplementary Material 1 [file 13046_2024_3152_MOESM1_ESM.docx]

***Supplementary Information***

***Supplementary Methods***

**Western blot analysis**

Protein samples from tissues and cells were obtained using RIPA lysis buffer. Samples were separated through 12.5% SDS-PAGE gels and nitrocellulose (NC) membranes were blocked with 5% skim milk at room temperature for 1 hour. Next, membranes were incubated overnight at 4°C with primary antibodies, as β-Tubulin (Proteintech, #10094-1-AP) was used as a loading control. The primary antibodies used in this study were obtained as follows: USP15 (CST, #66310), HKDC1 (Proteintech, #25874-1-AP), IGF2BP3 (Proteintech, #14642-1-AP), Flag-tag (Proteintech, # 66008-4-Ig), EMT antibody sampler kits (including E-cadherin, N-cadherin and Vimentin) (CST, #9782) and mitophagy antibody sampler kits (including SQSTM1, Parkin, PINK1 and LC3B) (CST, #43110). The secondary antibodies were obtained as follows: IRDye 800CW goat anti-mouse (LI-COR Biosciences, #926-32210) and donkey anti-rabbit (LI-COR Biosciences, #926-32213). The immunoreactivity was detected using Odyssey Infrared Imaging System (Gene Company Co. Ltd, Shanghai, China). The bands were quantified by measuring the band density.

**Co-immunoprecipitation (CoIP)**

CoIP assay was performed as previously described. Briefly, cells were lysed in IP buffer (Nonidet P40, 50 mM Tris-HCl [pH = 7.4], 50 mM EDTA, 150 mM NaCl and protease inhibitor) and centrifuged at 14,000 × g at 4 °C for 10 min. Next, supernatants were collected and incubated with USP15, HKDC1, IGF2BP3, and Flag-tag antibody at 4 °C for 2 hours, and then mixed with protein A/G agarose beads overnight at 4 °C on a rotating wheel. Beads were washed five times with IP buffer at 1,000 ×g for 5 min at 4 °C. Immunoprecipitants were boiled in 2×SDS loading buffer and then subjected to western blot analysis; 10% whole cell lysates were used as the loading control (10% Input).

**RNA extraction and qRT-PCR**

Total RNA was extracted from tissue samples and cell lines using TRIzol Reagent (Invitrogen, USA) according to the manufacturer’s instructions. First-strand cDNA was generated by RT-PCR using a reverse transcription system kit (Invitrogen, USA). qRT-PCR was performed with ABI PRISM 7500 Sequence Detection System according to the SYBR Green method. All reactions were performed at least three times. Cycle threshold (CT) values were determined using fixed threshold settings after completion of the reaction. mRNA levels of targets were normalized to those of TUBB. Their relative expression levels normalized to the control were further calculated using the 2^ΔΔ-ct^ method. The primers used are listed in Supplementary Table S3.

**Colony formation assay**

For colony formation assay, SGC7901 and BGC823 cells were seeded into 6-well plates at 600 cells/well and incubated at 37°C with 5% CO_2_ for two weeks. Then, GC cells were washed three times with PBS and fixed with methanol for 15 minutes at room temperature. The cells were stained with 0.1% crystal violet, and only colonies containing >100 cells were counted.

**EdU staining and cell proliferation assay**

SGC7901 and BGC823 cells were treated as indicated, washed three times with PBS, and incubated in serum-free DMEM containing 10 μmol/L EdU (RiboBio, Co. Ltd, Guangzhou, China) for 2 hours. Cells were fixed and stained according to the manufacturer’s instructions. Finally, cells were imaged by confocal microscope. The percentage of proliferating cells was further calculated.

To further determine the level of cell proliferation, cells were seeded into 96-well plates at a density of 3×10^3^/well, then monitored with IncuCyte live cell analysis imaging system (Essen Biosciences, USA).

**AnnexinV-FITC/PI analysis and TUNEL staining assay**

Cells were harvested and washed once with PBS, then resuspended with 1× Binding Buffer and labeled using Annexin-V-FITC and propidium iodide (PI) from the Annexin-V-FITC detection kit purchased from Dojindo Laboratories (#AD10, Kumamoto, Japan) according to the manufacturer’s protocol. The labeled cells were analyzed by BD Accuri C6 flow cytometer (BD, Biosciences, San Jose, CA, USA). To further detect DNA fragmentation and consequently dead cells, TUNEL staining was performed to identify dead cells by using In situ Cell Death Detection Kit (#11684795910, Roche, Mannheim, Germany).

**Cell migration and invasion assay**

The capacity of GC cells migration and invasion were tested using transwell chambers (8 μm pore size membranes). Lower chambers were added DMEM with 10% FBS and incubated at 37°C with 5% CO_2_. Then the upper surfaces were covered with matrigel (BD Biosciences, USA) for cell invasion while cell migration assay was conducted without matrigel. After the transfection of GC cells for 36 hours, these cells were seeded in upper chamber with serum-free medium. After 48 hours, cells were fixed with methanol and stained with 0.1% crystal violet. Images of cells were obtained through a microscope, and the numbers of migrated or invaded cells were counted by Image Pro Plus.

**Measurement of mitochondrial membrane potential**

The mitochondrial membrane potential of GC cells was measured using the sensitive and relatively mitochondrion specific lipophilic cationic probe JC-1 (#C2006, Beyotime Biotechnology, Shanghai, China). Briefly, GC cells cultured in DMEM were incubated with JC-1 (5 μmol/L) at 37°C for 20 min and then examined using flow cytometry (BD Accuri™ C6) or confocal microscope (Carl Zeiss, LSM 780). The intensities of green (excitation/emission wavelength = 485/538 nm) and red (excitation/emission wavelength = 485/590 nm) fluorescence were analyzed for ≥10 microscopic fields in each sample and represented a surrogate marker of loss of mitochondrial membrane potential.

**Intracellular ROS detection**

DCFH-DA (#S0033S, Beyotime Biotechnology, Shanghai, China) was used to detect intracellular ROS production because the fluorescence of this cell-permeable agent significantly increases after oxidation. GC cells cultured in DMEM were incubated with DCFH-DA (5 μmol/L) at 37°C for 30 min and then examined using flow cytometry (BD Accuri™ C6).

**Transmission electron microscopy**

Transmission electron microscopy (TEM) was used to investigate the changes in subcellular ultrastructure of GC cells treated with piceatannol. Samples were centrifuged at 4,000 rpm for 10 min, and pellets were collected and analyzed by electron microscopy. Pellets were fixed overnight in 2% glutaraldehyde in cacodylate buffer (0.1 M sodium cacodylate, 2 mM MgCl_2_) at 4 °C. After washing three times with cacodylate buffer at 4 °C, samples were post-fixed in 2% osmium tetroxide for 1 hour at 4 °C and rinsed with deionized water, then dehydrated through a gradient series of ethanol, ranging from 50 to 100% ethanol, 20 min at each step. Samples were then incubated with progressively concentrated propylene oxide dissolved in ethanol followed by infiltration with increasing concentration of Eponate 812 resin. Samples were baked in a 65 °C oven overnight and sectioned using an Ultra microtome. Sections were viewed with a Field Emission TEM unit (JEM-1400PLUS).

**Extracellular acidification rate and oxygen consumption rate assays**

The extracellular acidification rate (ECAR) and cellular oxygen consumption rate (OCR) were measured using Seahorse XF Glycolysis Stress Test Kit (Agilent Technologies, Palo Alto, CA, USA) and Seahorse XF Cell Mito Stress Test Kit (Agilent Technologies) and were performed using the Seahorse XF24 analyzer. USP15 knockdown or negative control GC cells were selected to detect glucose uptake. Briefly, cells were harvested at 48 hours after seeding, which were used to measure ECAR and OCR. After baseline measurements, for ECAR, the Seahorse automatically filled each well with 10 mmol/L glucose, 1 μmol/L oligomycin (the oxidative phosphorylation inhibitor), and 50 mmol/L 2-DG (2-deoxy-D-glucose, the glycolytic inhibitor) successively. For OCR, 1 μmol/L oligomycin, 1 μmol/L FCCP (p-trifluoromethoxy carbonyl cyanide phenylhydrazone, the reversible inhibitor of oxidative phosphorylation), and 0.5 μmol/L Rotenone were automatically injected successively. Data were analyzed by using Seahorse XF24 Wave software. ECAR in mpH/min and OCR in pmol/min are reported.

***Supplementary Data***


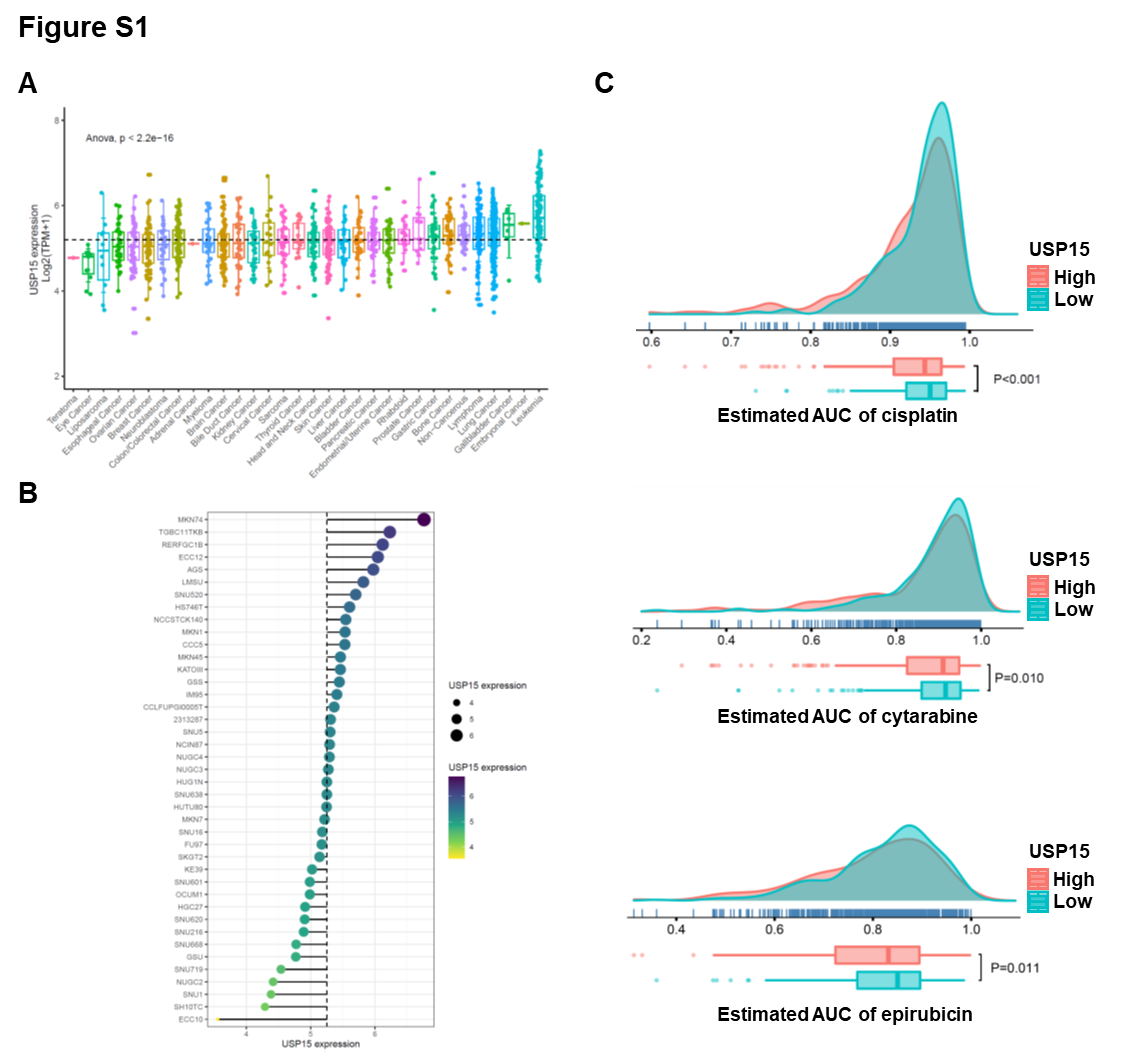


**Figure S1. (A)** USP15 mRNA expression level in multiple cancer tissues. Analysis of The Cancer Genome Atlas (TCGA) data from GDC portal. **(B)** USP15 mRNA expression level in several GC cell lines. Analysis of Cancer Cell Line Encyclopedia (CCLE) data from Broad DepMap portal. **(C)** Drug sensitivity analysis of USP15 expression status for the CCLE data based on the pRRophetic.


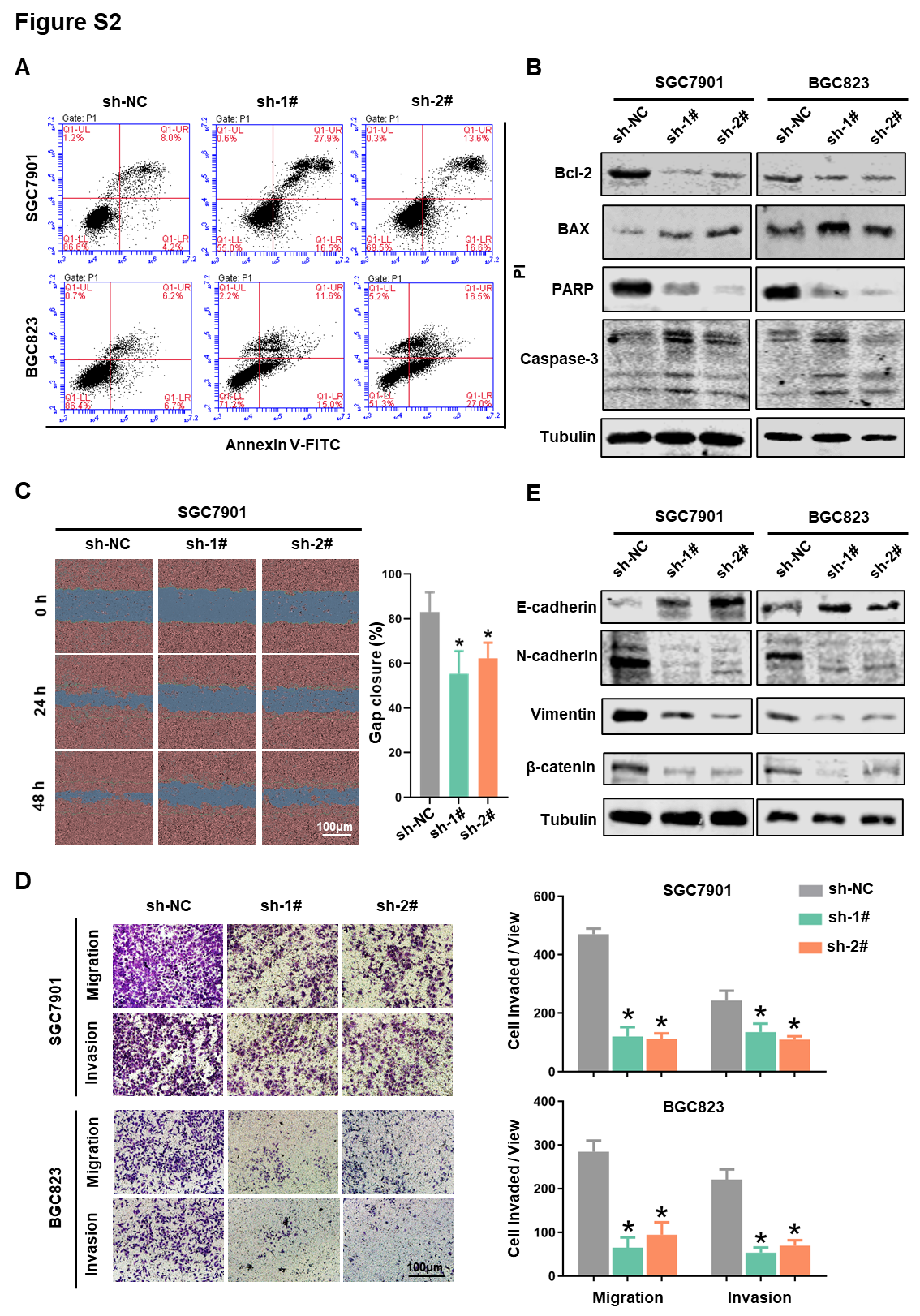


**Figure S2. (A)** Representative FCM plots using Annexin-V/PI staining for detecting cell death. **(B)** Western blot analysis presenting changes in the expression levels of Bcl-2, Bax, PARP, as well as the total and cleaved forms of Caspase-3.β-Tubulin was used as an internal control. **(C)** Wound-healing assay was used to determine the mobility of GC cells. **(D)** GC cells were further assayed for their invasive capability with or without matrigel on transwell chambers. Scale bar, 100 μm. **(E)** Western blot analysis of EMT-related proteins E-Cadherin, N-Cadherin and Vimentin after 48 hours’ co-transfection. α/β-Tublin was used as an internal control. Data are expressed as mean ± SD. **P* < 0.05 *vs.* sh-NC, n = 6 independent experiments.


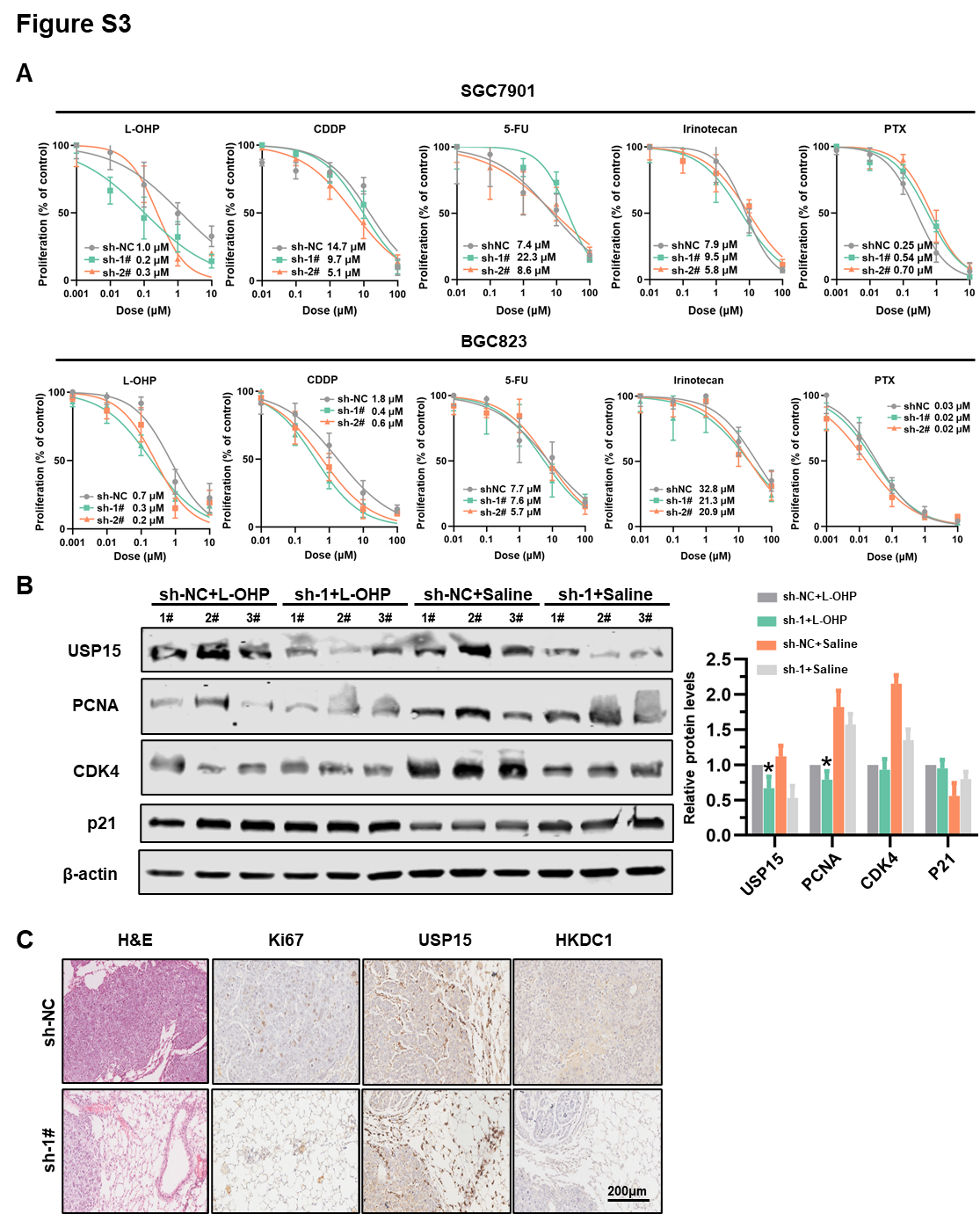


**Figure S3. (A)** GC cell proliferation among chemotherapy drugs was assayed using the IncuCyte system. The growth-inhibitory curves and IC_50_ of each group were further calculated using GraphPad software. L-OHP, Oxaliplatin; CDDP, Cisplatin; 5-FU, 5-Fluorouracil. **(B)** Western blot analysis of USP15, PCNA, CDK4, p21 in xenograft tissues. PCNA, Proliferating cell nuclear antigen; CDK4 and p21 (CDKN1A) are key regulators of cell cycle. β-actin was used as an internal control. Data are expressed as mean ± SD. **P* < 0.05 *vs.* sh-NC+L-OHP, n = 4 independent experiments. **(C)** The HE staining and the analysis of Ki67, USP15, and HKDC1 by IHC staining in lung tissues in a distal colonization model.


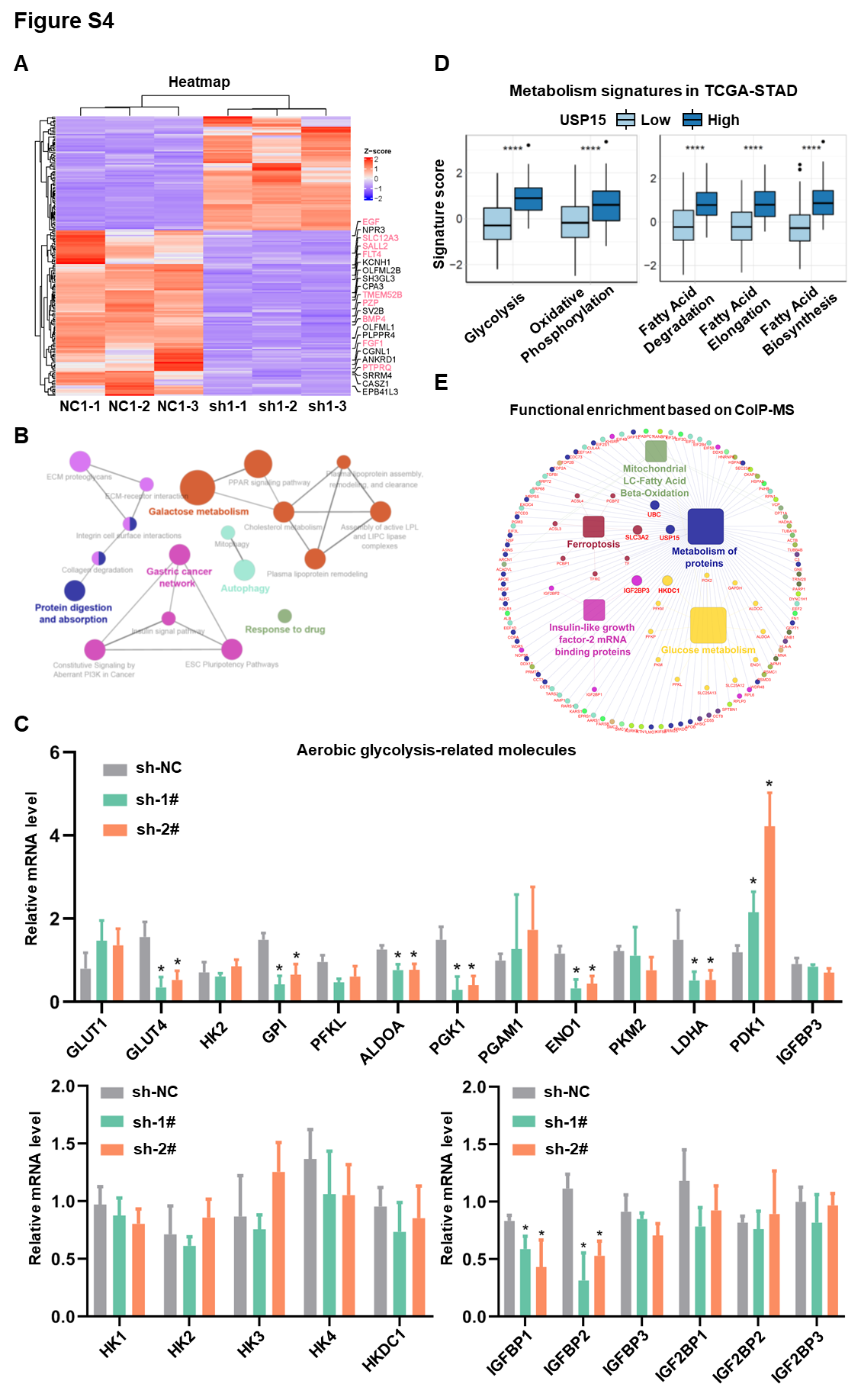


**Figure S4. (A)** The analysis of differentially expressed genes between SGC7901-shUSP15 group and negative control group based on RNAseq assay. The results showed downregulated expression of oncogenes, such as EGF, SLC12A3, and FGF1 in USP15 knockdown group. **(B)** The significant different expression genes (top 200) were further performed functional enrichment analysis by using WikiPathways dataset. The result showed that knockdown of USP15 significantly affected the expression network of pathogenic genes in GC and reshaped regulatory pathways, such as those involved in protein digestion, response to drug, and galactose metabolism. **(C)** qRT-PCR was used to detect the mRNA levels of metabolic-related genes. TUBB was used as an internal control. Data are expressed as mean ± SD. **P* < 0.05 *vs.* sh-NC, n = 6 independent experiments. **(D)** The comparative analysis of the differences between metabolism signatures for distinct USP15 expression levels using TCGA-STAD cohort. ****P* < 0.001 *vs.* USP15 low expression group. **(E)** Functional enrichment of protein-protein interactions obtained from CoIP-MS analysis using the STRING database.


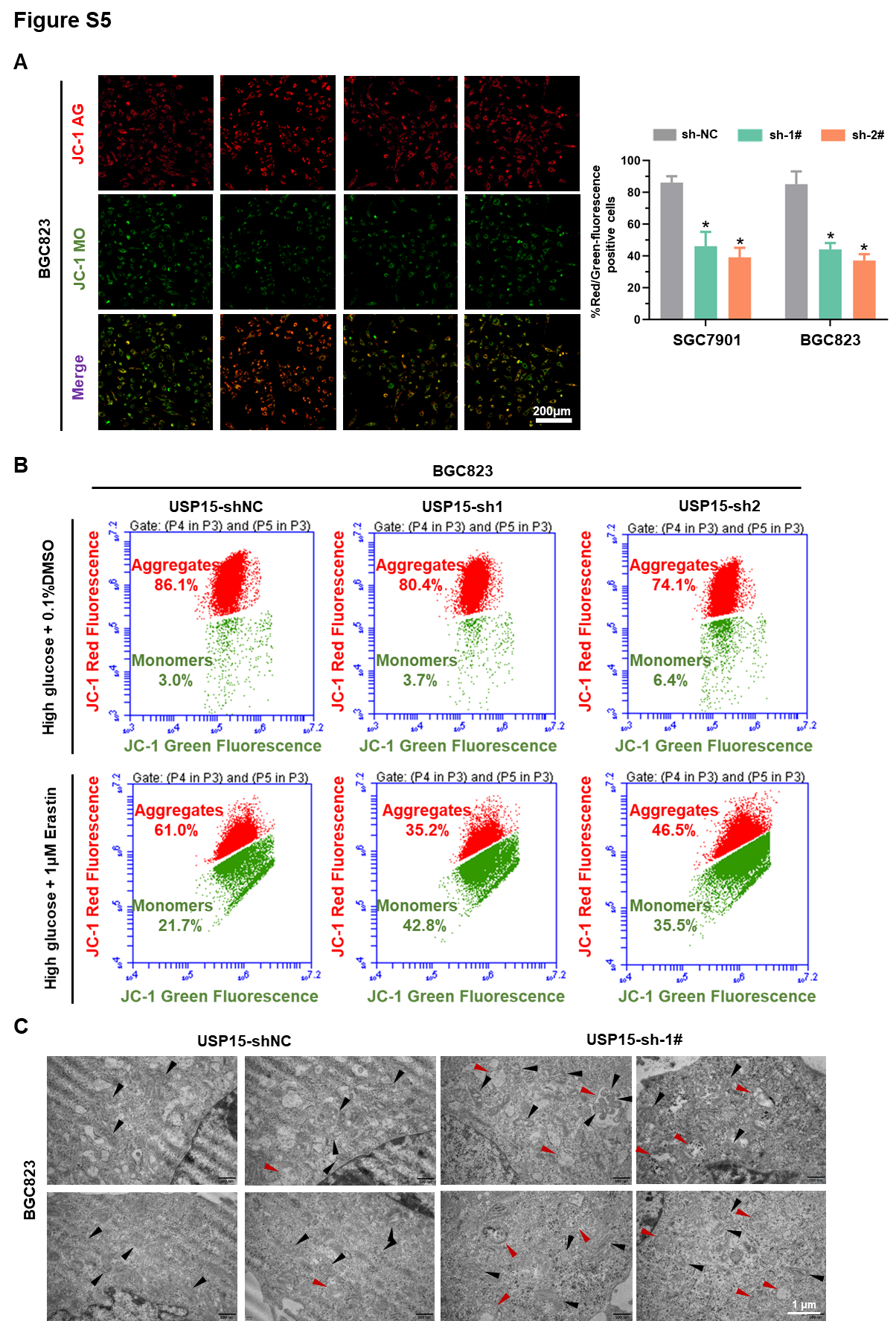


**Figure S5. (A)** Mitochondrial membrane potential of both USP15-knockdown and negative control BGC823 cells were detected by JC-1 probe. The red fluorescence represents the mitochondrial aggregate JC-1 and the green fluorescence indicates the monomeric JC-1. Scale bar = 200 μm. Representative images were shown in the left and the percentage of positive cells was further counted in the right. Data are expressed as mean ± SD. **P* < 0.05 *vs.* sh-NC, n = 6 independent experiments. **(B)** Changes of mitochondrial membrane potential were further measured quantitatively by FACS analysis. Erastin was used to trigger the mitochondrial stress in GC cells. **(C)** Representative transmission electron microscope (TEM) images of the morphological and subcellular structure of different groups. Red arrows represent mitochondria with abnormal structure, and black arrow represent mitophagy. Scale bar = 1 μm.


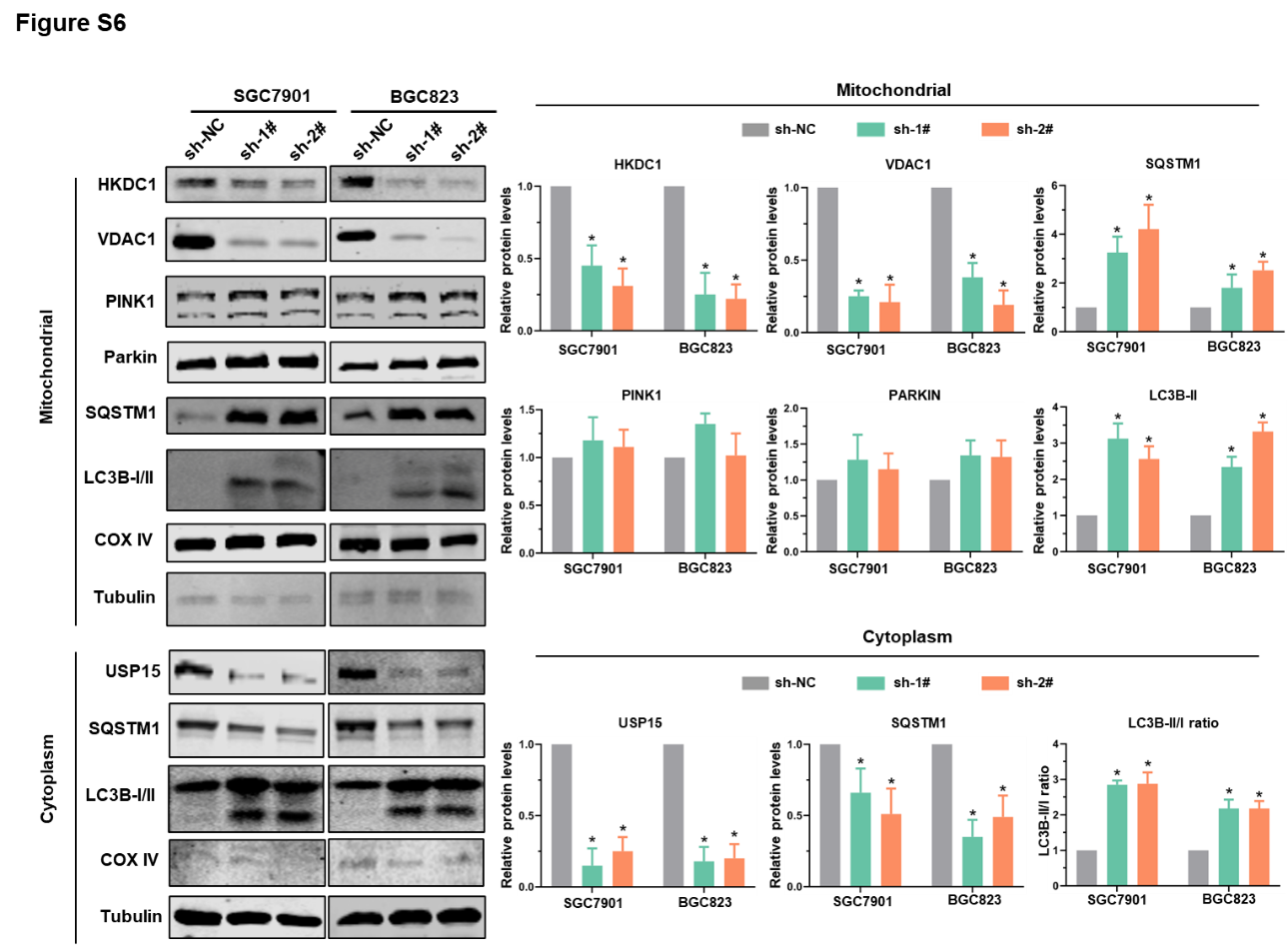


**Figure S6.** Cytoplasmic and mitochondrial levels of HKDC1, VDAC1, PINK1, Parkin, SQSTM1, LC3B-I/II, and USP15 were determined by western blot. COX IV was used as an internal control for mitochondrial proteins, α/β-Tublin was used as an internal control for cytoplasmic proteins. The quantitative protein expression levels are shown on the right. Data are expressed as mean ± SD. **P* < 0.05 *vs.* sh-NC, n = 6 independent experiments.


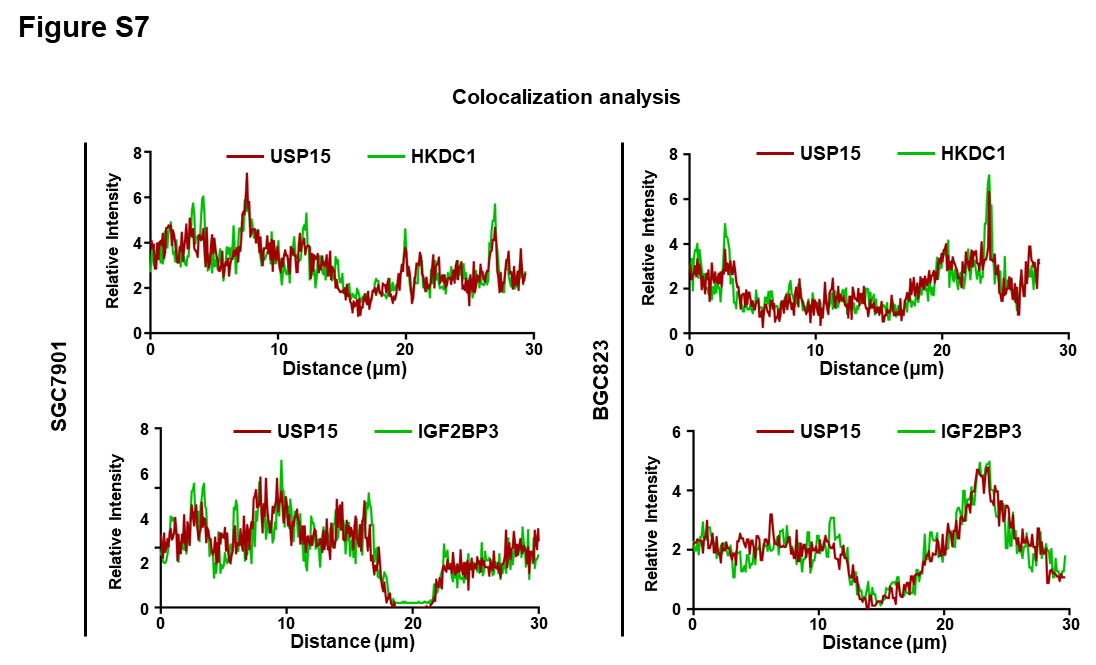


**Figure S7** **Colocalization analysis between USP15 and HKDC1 in GC cells based on dual-color confocal imaging.** The correlation results are derived from Figure 6C.

**
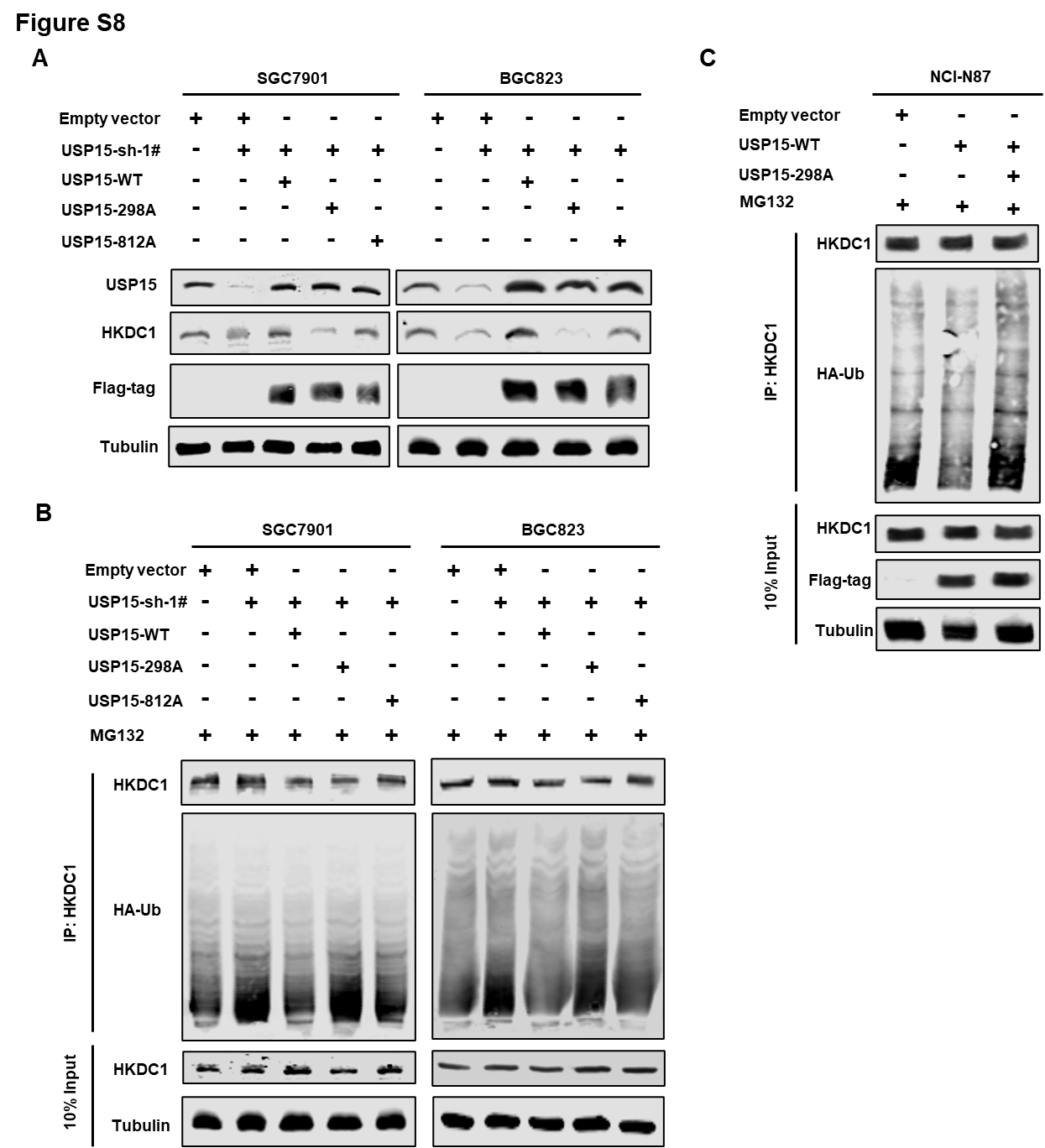
**

**Figure S8** We constructed expression plasmids for several recombinant USP15 proteins fused with Flag tags, including wild-type (USP15-WT), enzyme-activity-deficient (USP15-298A), and polyubiquitination-recognition-deficient (USP15-812A), and transfected GC cells with these plasmids. **(A)** Western blot showed expression levels of USP15, HKDC1 and flag-tag in these cells. β-Tubulin was used as an internal control. **(B)** The ubiquitinated reporter plasmid with HA tag (HA-Ub) was transfected into the above cells, respectively. Immunoprecipitation experiments were performed using anti-HKDC1 antibody. The ubiquitination level of HKDC1 was further detected by western blot using anti-HA antibody. MG132 (5uM, 4h) is a proteasome inhibitor. **(C)** Likewise, we transfected USP15 wild-type (USP15-WT) and mutant (USP15-298A) expression plasmids in NCI-N87 cells, respectively.


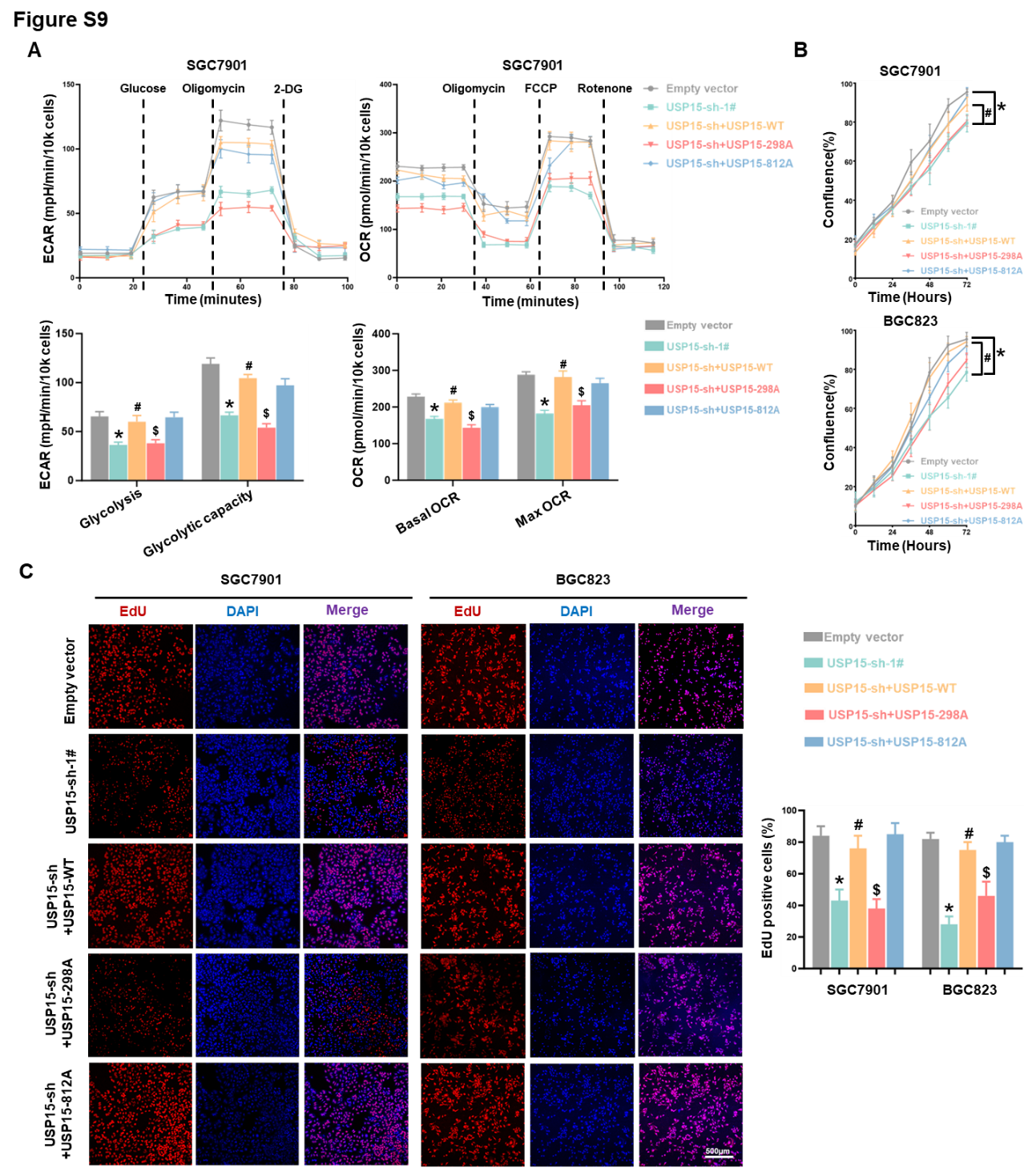


**Figure S9 (A)** Both ECAR and OCR of stable USP15-knockdown, empty vector, USP15-WT, USP15-298A and USP15-812A of SGC7901 cells were measured. For ECAR, the Seahorse automatically filled each well with 10 mmol/L glucose, 1 µmol/L oligomycin, and 50 mmol/L 2-DG successively. Basal glycolysis was determined after addition of glucose, and glycolytic capacity was calculated after addition of oligomycin. For OCR, 1 µmol/L oligomycin, 1 µmol/L FCCP, and 0.5 µmol/L rotenone were automatically injected successively. Basal OCR was determined prior to addition of oligomycin, and maximal respiratory capacity was determined by subtracting nonmitochondrial OCR, calculated after rotenone injection to maximal OCR upon FCCP uncoupling and maximal electron transport. **(B)** Cell proliferation was measured by IncuCyte system. **(C)** The activity of DNA replication was detected by EdU staining. Scale bar = 500 μm. Data are expressed as mean ± SD. **P* < 0.05 *vs.* empty vector, #*P* < 0.05 *vs.* USP15-sh-1#, $*P* < 0.05 *vs.* USP15-sh+USP15-WT, n = 12 independent experiments.


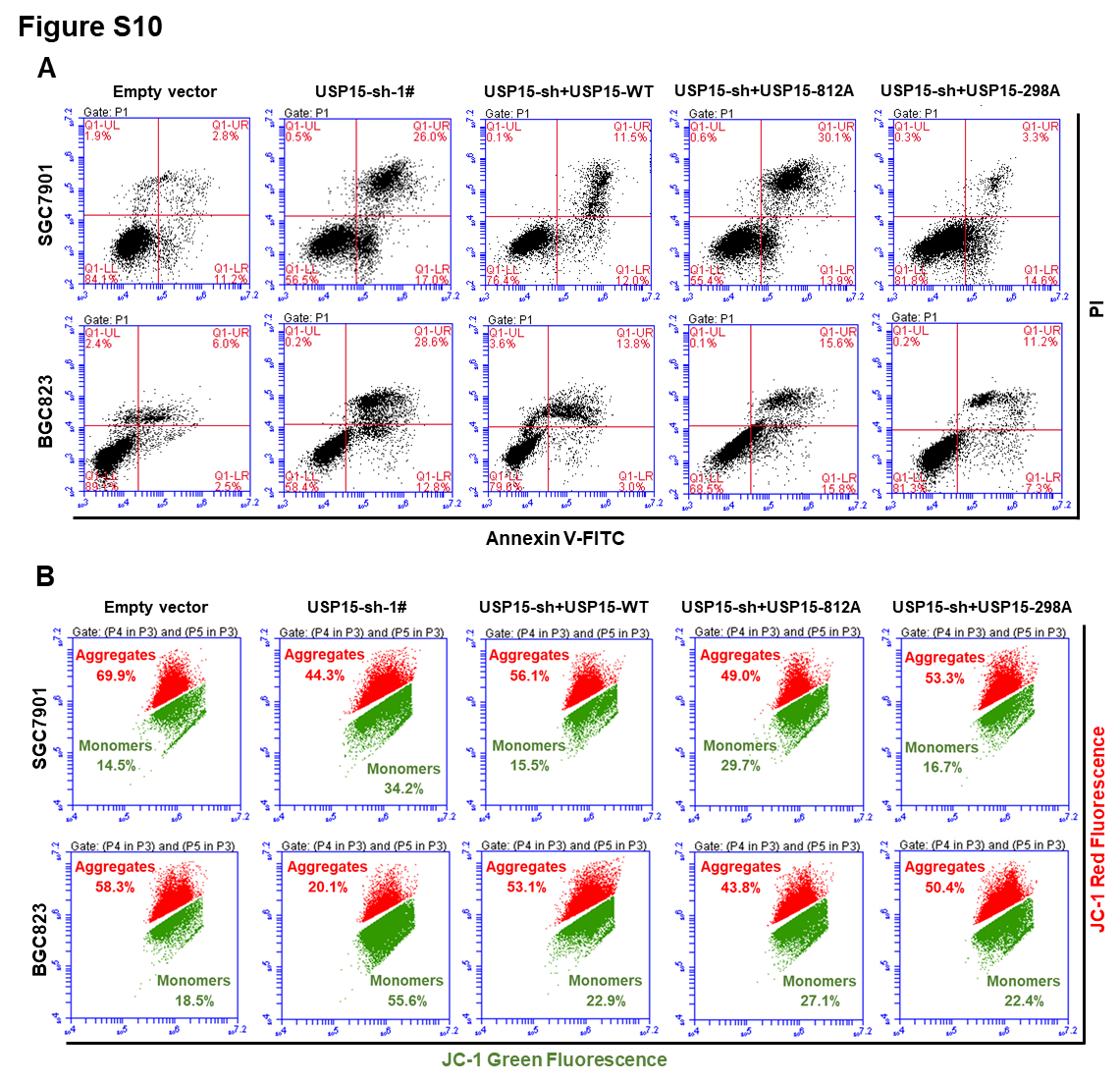


**Figure S10 (A)** Cell apoptosis was determined through FACS based on Annexin-V/PI staining method. **(B)** Changes of mitochondrial membrane potential were further measured quantitatively by FACS analysis. Erastin was used to trigger the mitochondrial stress in GC cells.


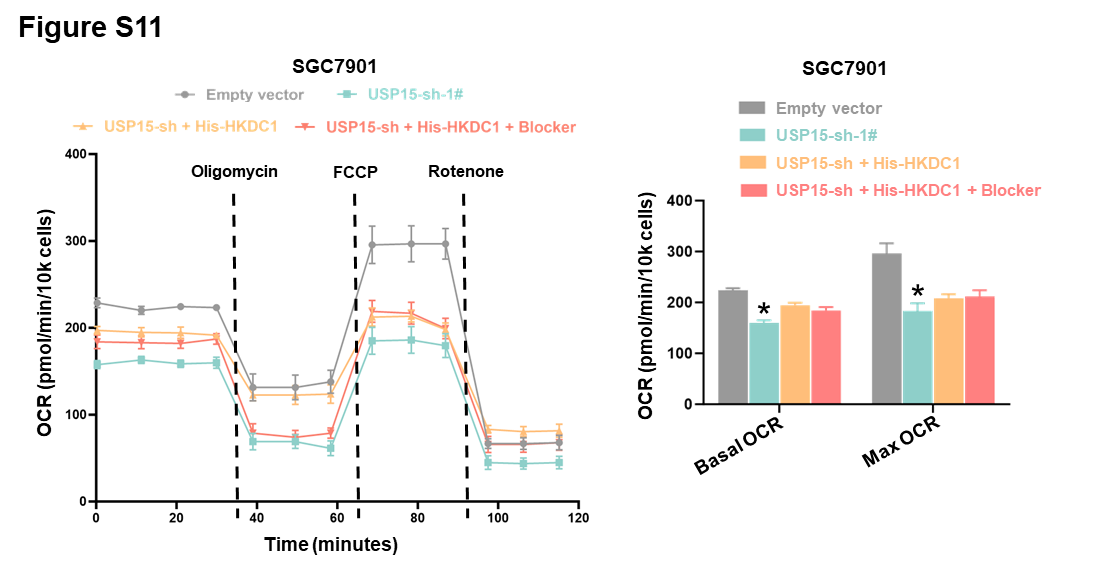


**Figure S11** OCAR of USP15-sh-1#, USP15-sh + His-HKDC1, USP15-sh + His-HKDC1 + blocker and empty vector cells were measured. Data are expressed as mean ± SD. **P* < 0.05 *vs.* empty vector, n = 12 independent experiments.


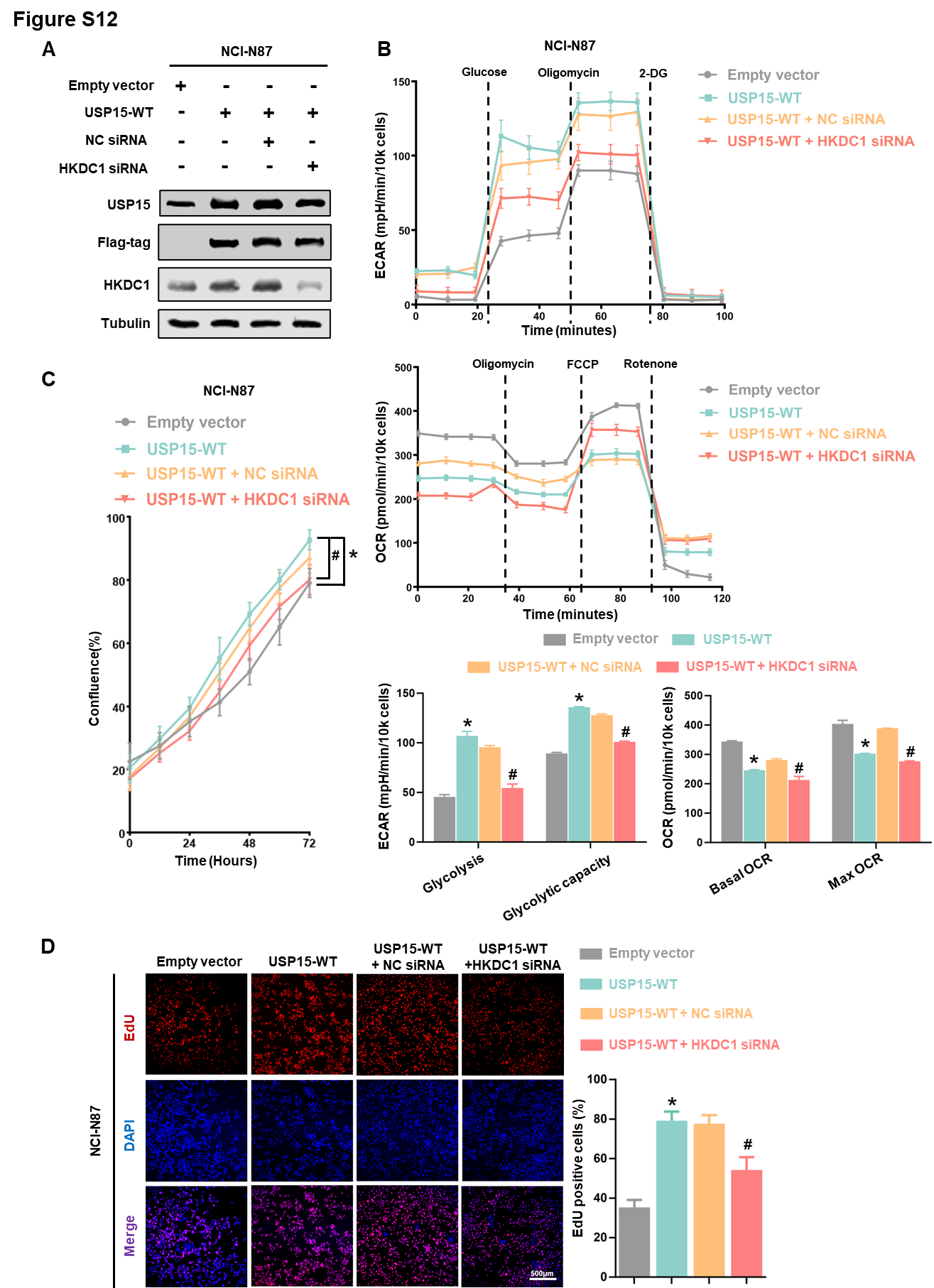


**Figure S12 (A)** Western blot analysis was performed to determine the expression levels of USP15, HKDC1 and flag-tag after transfected with HKDC1 siRNA in NCI-N87. α/β-Tublin was used as an internal control. **(B)** Both ECAR and OCAR of USP15-WT, USP15-WT + NC-siRNA, USP15-WT + HKDC1 siRNA and empty vector cells were measured. Data are expressed as mean ± SD. **P* < 0.05 *vs.* empty vector, #*P* < 0.05 *vs.* USP15-WT + NC-siRNA, n = 12 independent experiments. **(C)** Cell proliferation was measured by IncuCyte live cell. **(D)** The activity of DNA replication was detected by EdU staining. Scale bar = 500 μm. **P* < 0.05 *vs.* empty vector, #*P* < 0.05 *vs.* USP15-WT + NC-siRNA.

***Supplementary Tables***

**Table S1.** Pathological information of gastric tissue samples for USP15 IHC validation.

| Patient No | Sex | Age | pTNM | pStage | Lauren classification | Lauren classification |  |
| --- | --- | --- | --- | --- | --- | --- | --- |
| 1 | M | 56 | T2N0M0 | IB | Intestinal | Moderate |  |
| 2 | | F | 47 | T3N2M0 | IIIA | N/A | Moderate |
| 3 | | M | 59 | T3N3M0 | IIIB | Intestinal | Moderate |
| 4 | | M | 53 | T2N2M0 | IIB | Mixed | Poor |
| 5 | | M | 55 | T3N3M0 | IIIB | Diffuse | Well |
| 6 | | M | 56 | T3N2M0 | IIIA | Intestinal | Moderate |
| 7 | | F | 57 | T3N0M0 | IIA | Intestinal | Moderate |
| 8 | | F | 74 | T3N0M0 | IIA | Diffuse | Poor |
| 9 | | M | 58 | T3N0M0 | IIA | Intestinal | Moderate |
| 10 | | M | 55 | T4aN1M0 | IIIA | Diffuse | Poor |
| 11 | | M | 58 | T3N2M0 | IIIA | Intestinal | Moderate |
| 12 | | M | 78 | T2N1M0 | IIA | Mixed | Moderate |
| 13 | | F | 50 | T2N3M0 | IIIA | Intestinal | Moderate |
| 14 | | F | 32 | T3N3M0 | IIIB | Diffuse | Poor |
| 15 | | M | 68 | T3N3M0 | IIIB | Mixed | Moderate |
| 16 | | M | 41 | T3N2M0 | IIIA | Diffuse | Poor |
| 17 | | M | 69 | T3N3M0 | IIIB | Diffuse | Poor |
| 18 | | F | 60 | T3N2M1 | IV | Intestinal | Poor |
| 19 | | F | 41 | T3N2M0 | IIIA | Intestinal | Moderate |
| 20 | | F | 56 | T1N0M0 | IA | Mixed | Poor |
| 21 | | M | 75 | T3N1M0 | IIB | Mixed | Moderate |
| 22 | | M | 75 | T3N0M0 | IIA | Intestinal | Moderate |
| 23 | | M | 45 | T3N0M0 | IIA | Diffuse | Poor |
| 24 | | M | 55 | T1N0M0 | IA | Intestinal | Moderate |
| 25 | | M | 44 | T3N1M0 | IIB | Intestinal | Moderate |
| 26 | | M | 60 | T3N1M0 | IIB | Intestinal | Moderate |
| 27 | | F | 53 | T2N0M0 | IB | Mixed | Moderate |
| 28 | | F | 57 | T3N0M0 | IIA | Diffuse | Poor |
| 29 | | M | 38 | T3N3M0 | IIIB | Diffuse | Moderate |
| 30 | | F | 56 | T3N0M0 | IIA | Diffuse | Poor |
| 31 | | M | 62 | T2N0M0 | IB | Intestinal | Moderate |
| 32 | | M | 69 | T2N3M0 | IIIA | Intestinal | Moderate |
| 33 | | F | 48 | T2N0M0 | IB | Mixed | Moderate |
| 34 | | F | 70 | T2N1M0 | IIA | Mixed | Poor |
| 35 | | M | 80 | T3N2M0 | IIIA | Diffuse | Poor |
| 36 | | M | 65 | T3N1M0 | IIB | Mixed | Moderate |
| 37 | | M | 61 | T3N0M0 | IIA | Intestinal | N/A |
| 38 | | M | 59 | T3N3M0 | IIIB | Diffuse | Poor |
| 39 | | F | 53 | T3N3M0 | IIIB | Mixed | Poor |
| 40 | | F | 74 | T3N0M0 | IIA | Intestinal | Poor |
| 41 | | M | 61 | T2N2M0 | IIB | Intestinal | Poor |
| 42 | | M | 72 | T3N1M0 | IIB | Intestinal | Moderate |
| 43 | | M | 49 | T3N1M0 | IIB | Intestinal | Moderate |
| 44 | | F | 64 | T4aN3M0 | IIIB | Diffuse | Poor |
| 45 | | F | 58 | T1N0M0 | IA | Diffuse | Poor |
| 46 | | M | 71 | T2N1M0 | IIA | Intestinal | Poor |
| 47 | | M | 70 | T2N1M0 | IIA | Intestinal | N/A |
| 48 | | M | 63 | T1N0M0 | IA | Mixed | Poor |
| 49 | | M | 67 | T4aN2M0 | IIIB | Intestinal | Poor |
| 50 | | M | 54 | T4bN0M0 | IIIB | N/A | Poor |
| 51 | | M | 66 | T4aN3M0 | IIIB | N/A | Poor |
| 52 | | M | 56 | T3N0M0 | IIA | Mixed | Moderate |
| 53 | | F | 55 | T3N0M0 | IIA | Diffuse | Poor |
| 54 | | M | 45 | T1N0M0 | IA | Diffuse | Poor |
| 55 | | M | 52 | T3N3M0 | IIIB | Intestinal | Moderate |
| 56 | | M | 36 | T3N0M0 | IIA | Diffuse | Poor |
| 57 | | M | 60 | T4aN2M0 | IIIB | Diffuse | Poor |
| 58 | | M | 51 | T3N3M0 | IIIB | Intestinal | Poor |
| 59 | | F | 69 | T3N1M0 | IIB | Mixed | Moderate |
| 60 | | M | 59 | T3N3M0 | IIIB | Intestinal | Well |
| 61 | | M | 48 | T3N3M0 | IIIB | Diffuse | Poor |
| 62 | | M | 46 | T4aN1M0 | IIIA | Mixed | Poor |
| 63 | | F | 41 | T3N2M0 | IIIA | Mixed | Moderate |
| 64 | | M | 54 | T3N1M0 | IIB | Intestinal | Moderate |
| 65 | | M | 58 | T2N3M0 | IIIA | Diffuse | Poor |
| 66 | | M | 55 | T3N2M0 | IIIA | Diffuse | Poor |
| 67 | | M | 62 | T3N2M0 | IIIA | Intestinal | Moderate |
| 68 | | M | 51 | T2N0M0 | IB | Intestinal | Moderate |
| 69 | | F | 68 | T4aN2M0 | IIIB | Intestinal | Moderate |
| 70 | | M | 42 | T3N2M0 | IIIA | Intestinal | Moderate |
| 71 | | F | 53 | T3N2M0 | IIIA | Diffuse | Poor |
| 72 | | M | 52 | T3N1M0 | IIB | Intestinal | Moderate |
| 73 | | F | 34 | T2N0M0 | IB | N/A | Moderate |
| 74 | | M | 49 | T3N3M0 | IIIB | N/A | Moderate |
| 75 | | M | 71 | T3N0M0 | IIA | N/A | Moderate |
| 76 | | M | 61 | T2N2M0 | IIB | N/A | Moderate |
| 77 | | F | 58 | T3N2M0 | IIIA | N/A | Moderate |
| 78 | | F | 67 | T2N2M0 | IIB | Intestinal | Moderate |
| 79 | | M | 59 | T3N0M0 | IIA | Intestinal | Moderate |
| 80 | | F | 52 | T3N1M0 | IIB | Intestinal | Moderate |
| 81 | | M | 82 | T2N3M0 | IIIA | Diffuse | Poor |
| 82 | | F | 52 | T4aN1M0 | IIIA | Intestinal | Moderate |
| 83 | | F | 52 | T3N2M0 | IIIA | Intestinal | Moderate |
| 84 | | M | 69 | T3N0M0 | IIA | Intestinal | Moderate |
| 85 | | F | 79 | T2N0M0 | IB | Intestinal | Poor |
| 86 | | M | 58 | T3N3M0 | IIIB | Mixed | Poor |
| 87 | | F | 64 | T3N3M0 | IIIB | Mixed | Moderate |
| 88 | | M | 52 | T3N0M0 | IIA | Intestinal | Moderate |
| 89 | | M | 60 | T4aN2M0 | IIIB | N/A | Moderate |
| 90 | | F | 71 | T3N2M0 | IIIA | Mixed | Moderate |
| 91 | | F | 81 | T4bN3M1 | IV | Mixed | Moderate |
| 92 | | M | 74 | T2N1M0 | IIA | Intestinal | Moderate |
| 93 | | F | 62 | T2N3M0 | IIIA | Diffuse | Poor |
| 94 | | M | 47 | T2N0M0 | IB | Diffuse | Poor |
| 95 | | F | 71 | T2N0M0 | IB | Mixed | Poor |
| 96 | | M | 69 | T3N0M0 | IIA | Diffuse | Poor |
| 97 | | M | 63 | T3N3M0 | IIIB | Intestinal | Moderate |
| 98 | | M | 57 | T3N3M0 | IIIB | Mixed | Moderate |
| 99 | | M | 50 | T3N1M0 | IIB | Mixed | Moderate |
| 100 | | M | 57 | T3N2M0 | IIIA | Mixed | Poor |
| 101 | | M | 64 | T4aN2M0 | IIIB | Intestinal | Moderate |
| 102 | | M | 71 | T2N0M0 | IB | Mixed | Poor |
| 103 | | M | 33 | T3N2M0 | IIIA | Mixed | Poor |
| 104 | | M | 69 | T3N0M0 | IIA | Intestinal | Moderate |
| 105 | | M | 56 | T3N3M0 | IIIB | Intestinal | Moderate |
| 106 | | M | 42 | T3N0M0 | IIA | Mixed | Moderate |
| 107 | | F | 64 | T3N3M0 | IIIB | Intestinal | Moderate |
| 108 | | M | 58 | T3N3M1 | IV | Mixed | N/A |
| 109 | | M | 67 | T3N3M1 | IV | Intestinal | Poor |
| 110 | | F | 72 | T3N3M1 | IV | Intestinal | Poor |
| 111 | | F | 74 | T4bN3M0 | IIIC | Intestinal | Moderate |
| 112 | | M | 65 | T4bN3M0 | IIIC | Intestinal | Moderate |
| 113 | | F | 65 | T4bN2M0 | IIIC | Intestinal | Moderate |
| 114 | | F | 67 | T3N3M0 | IIIB | Intestinal | Moderate |
| 115 | | M | 70 | T3N3M0 | IIIB | Mixed | Well |
| 116 | | M | 68 | T3N3M0 | IIIB | Mixed | Well |
| 117 | | M | 68 | T3N3M0 | IIIB | Mixed | Well |
| 118 | | M | 52 | T3N3M0 | IIIB | Diffuse | Moderate |
| 119 | | M | 65 | T3N3M0 | IIIB | Diffuse | Moderate |
| 120 | | F | 59 | T3N3M0 | IIIB | Diffuse | Moderate |
| 121 | | M | 63 | T3N3M0 | IIIB | Intestinal | Moderate |
| 122 | | F | 68 | T3N3M0 | IIIB | Intestinal | Moderate |
| 123 | | M | 52 | T3N3M0 | IIIB | Intestinal | Moderate |
| 124 | | M | 68 | T3N3M0 | IIIB | Mixed | Poor |
| 125 | | M | 84 | T4aN1M0 | IIIA | Intestinal | Poor |
| 126 | | M | 60 | T3N2M0 | IIIA | Intestinal | Moderate |
| 127 | | M | 69 | T3N2M0 | IIIA | Diffuse | Poor |
| 128 | | M | 77 | T3N2M0 | IIIA | Intestinal | Poor |
| 129 | | M | 75 | T3N2M0 | IIIA | Intestinal | Poor |
| 130 | | F | 70 | T3N2M0 | IIIA | Intestinal | Poor |
| 131 | | M | 48 | T3N2M0 | IIIA | Intestinal | Poor |
| 132 | | F | 68 | T3N2M0 | IIIA | Intestinal | Poor |
| 133 | | F | 65 | T3N2M0 | IIIA | Mixed | Poor |
| 134 | | M | 53 | T3N2M0 | IIIA | Mixed | Poor |
| 135 | | F | 51 | T3N2M0 | IIIA | Diffuse | Poor |
| 136 | | M | 56 | T3N2M0 | IIIA | Diffuse | Poor |
| 137 | | M | 83 | T3N2M0 | IIIA | Intestinal | Moderate |
| 138 | | F | 43 | T3N2M0 | IIIA | Intestinal | Moderate |
| 139 | | F | 54 | T3N2M0 | IIIA | Intestinal | Moderate |
| 140 | | M | 58 | T3N1M0 | IIB | Intestinal | Well |
| 141 | | F | 84 | T3N1M0 | IIB | Intestinal | Well |
| 142 | | M | 71 | T3N1M0 | IIB | Intestinal | Well |
| 143 | | M | 65 | T3N1M0 | IIB | Intestinal | Well |
| 144 | | M | 24 | T4bN0M0 | IIIB | Intestinal | Moderate |
| 145 | | M | 65 | T3N1M0 | IIB | Mixed | Moderate |
| 146 | | M | 67 | T3N1M0 | IIB | Intestinal | Moderate |
| 147 | | M | 65 | T3N0M0 | IIA | Intestinal | Moderate |
| 148 | | M | 60 | T3N0M0 | IIA | Intestinal | Moderate |
| 149 | | M | 70 | T3N0M0 | IIA | Intestinal | Moderate |
| 150 | | M | 53 | T3N0M0 | IIA | Intestinal | Moderate |
| 151 | | M | 78 | T3N0M0 | IIA | Intestinal | Moderate |
| 152 | | M | 61 | T3N0M0 | IIA | Intestinal | Moderate |
| 153 | | M | 73 | T2N1M0 | IIA | Diffuse | Moderate |
| 154 | | F | 68 | T3N0M0 | IIA | Diffuse | Well |
| 155 | | M | 70 | T3N0M0 | IIA | Diffuse | Well |
| 156 | | F | 47 | T2N1M0 | IIA | Diffuse | Well |
| 157 | | F | 75 | T2N1M0 | IIA | Intestinal | Well |
| 158 | | M | 59 | T2N0M0 | IB | Intestinal | Moderate |
| 159 | | F | 70 | T1N0M0 | IA | Intestinal | Poor |
| 160 | | F | 73 | T1N0M0 | IA | Intestinal | Well |
| 161 | | F | 68 | T3N2M0 | IIIA | Intestinal | Moderate |
| 162 | | M | 61 | T3N2M0 | IIIA | Intestinal | Moderate |
| 163 | | F | 53 | T4bN2M0 | IIIC | Diffuse | Moderate |
| 164 | | M | 76 | T3N0M0 | IIA | Intestinal | Well |
| 165 | | M | 57 | T3N3M0 | IIIB | Intestinal | Moderate |
| 166 | | F | 79 | T1N0M0 | IA | N/A | N/A |
| 167 | | M | 67 | T1N0M0 | IA | N/A | N/A |
| 168 | | M | 70 | T3N2M0 | IIIA | Diffuse | Moderate |
| 169 | | M | 45 | T2N2M0 | IIB | Mixed | Moderate |
| 170 | | F | 30 | T3N3M0 | IIIB | Mixed | Moderate |
| 171 | | M | 67 | T2N3M0 | IIIA | Intestinal | Moderate |
| 172 | | M | 58 | T1N0M0 | IA | N/A | Well |
| 173 | | M | 47 | T2N1M0 | IIA | N/A | Well |
| 174 | | F | 50 | T2N1M0 | IIA | N/A | Moderate |
| 175 | | M | 59 | T1N0M0 | IA | N/A | Well |
| 176 | | F | 58 | T2N1M0 | IIA | N/A | Well |
| 177 | | F | 68 | T2N3M0 | IIIA | Intestinal | Moderate |
| 178 | | M | 57 | T1N0M0 | IA | Intestinal | Moderate |
| 179 | | M | 50 | T3N2M0 | IIIA | Intestinal | Moderate |
| 180 | | M | 58 | T3N1M0 | IIB | Diffuse | Moderate |
| 181 | | F | 45 | T2N3M0 | IIIA | Mixed | Moderate |
| 182 | | F | 32 | T2N0M0 | IB | Intestinal | Moderate |
| 183 | | M | 79 | T4bN3M0 | IIIC | Intestinal | Poor |
| 184 | | F | 59 | T1N0M0 | IA | Mixed | Moderate |
| 185 | | M | 53 | T2N1M0 | IIA | Diffuse | Moderate |
| 186 | | F | 42 | T2N1M0 | IIA | Diffuse | Moderate |
| 187 | | F | 53 | T3N1M0 | IIB | Intestinal | Poor |
| 188 | | F | 50 | T2N0M0 | IB | Intestinal | Well |
| 189 | | F | 55 | T3N3M0 | IIIB | Intestinal | Poor |
| 190 | | F | 72 | T2N3M0 | IIIA | Intestinal | Moderate |
| 191 | | F | 69 | T1N0M0 | IA | Intestinal | Well |
| 192 | | F | 33 | T4bN3M0 | IIIC | Intestinal | Poor |
| 193 | | M | 55 | T4bN0M0 | IIIB | Diffuse | Moderate |
| 194 | | F | 41 | T3N0M0 | IIA | Intestinal | Moderate |
| 195 | | M | 49 | T2N0M0 | IB | N/A | Well |
| 196 | | M | 36 | T3N2M0 | IIIA | Intestinal | Moderate |
| 197 | | M | 58 | T3N2M0 | IIIA | Intestinal | Moderate |
| 198 | | M | 32 | T4bN3M0 | IIIC | Intestinal | Moderate |
| 199 | | M | 62 | T2N0M0 | IB | N/A | N/A |
| 200 | | M | 60 | T3N3M1 | IV | Mixed | Poor |
| 201 | | M | 58 | T4aN3M1 | IV | Mixed | Poor |
| 202 | | F | 45 | T3N3M1 | IV | Intestinal | Moderate |
| 203 | | F | 52 | T3N1M1 | IV | Intestinal | Moderate |
| 204 | | F | 55 | T4bN3M1 | IV | Diffuse | Moderate |
| 205 | | F | 81 | T4bN3M1 | IV | Diffuse | Moderate |
| 206 | | F | 60 | T3N2M1 | IV | Intestinal | Poor |
| 207 | | M | 57 | T3N3M1 | IV | Mixed | Moderate |
| 208 | | F | 54 | T4aN2M1 | IV | Diffuse | Moderate |
| 209 | | F | 77 | T4aN3M1 | IV | N/A | Moderate |
| 210 | | F | 52 | T3N2M1 | IV | N/A | Moderate |
| 211 | | M | 79 | T3N3M0 | IIIB | Intestinal | Moderate |
| 212 | | M | 54 | T3N1M0 | IIB | Intestinal | Well |
| 213 | | M | 52 | T3N1M0 | IIB | Intestinal | Moderate |
| 214 | | F | 52 | T4aN1M0 | IIIA | Intestinal | Moderate |
| 215 | | F | 64 | T3N3M0 | IIIB | Mixed | Moderate |
| 216 | | M | 66 | T2N0M0 | IB | Intestinal | Moderate |
| 217 | | M | 79 | T3N2M0 | IIIA | Diffuse | Poor |
| 218 | | M | 57 | T3N2M0 | IIIA | Mixed | Poor |
| 219 | | M | 56 | T2N0M0 | IB | Intestinal | Moderate |
| 220 | | F | 57 | T3N0M0 | IIA | Intestinal | Moderate |
| 221 | | M | 41 | T3N2M0 | IIIA | Diffuse | Poor |
| 222 | | F | 56 | T1N0M0 | IA | Mixed | Poor |
| 223 | | M | 80 | T3N3M0 | IIIB | Intestinal | Moderate |
| 224 | | M | 69 | T2N3M0 | IIIA | Intestinal | Moderate |
| 225 | | M | 61 | T3N0M0 | IIA | Intestinal | N/A |
| 226 | | M | 59 | T3N3M0 | IIIB | Intestinal | Poor |
| 227 | | M | 45 | T1N0M0 | IA | Diffuse | Poor |
| 228 | | M | 58 | T2N3M0 | IIIA | Diffuse | Poor |
| 229 | | M | 55 | T3N2M0 | IIIA | Diffuse | Poor |
| 230 | | M | 62 | T3N2M0 | IIIA | Intestinal | Moderate |
| 231 | | M | 51 | T2N0M0 | IB | Intestinal | Well |
| 232 | | F | 68 | T4bN2M0 | IIIC | Intestinal | Moderate |
| 233 | | M | 42 | T3N2M0 | IIIA | Intestinal | Moderate |
| 234 | | F | 53 | T3N2M0 | IIIA | Diffuse | Poor |
| 235 | | F | 34 | T2N0M0 | IB | N/A | Moderate |
| 236 | | M | 49 | T3N3M0 | IIIB | N/A | Moderate |
| 237 | | M | 71 | T3N0M0 | IIA | N/A | Moderate |
| 238 | | M | 75 | T3N2M0 | IIIA | N/A | Moderate |
| 239 | | M | 61 | T2N2M0 | IIB | N/A | Moderate |
| 240 | | F | 58 | T3N2M0 | IIIA | N/A | Moderate |
| 241 | | F | 67 | T2N2M0 | IIB | Intestinal | Moderate |
| 242 | | M | 59 | T3N0M0 | IIA | Intestinal | Moderate |
| 243 | | F | 52 | T3N1M0 | IIB | Intestinal | Moderate |
| 244 | | M | 82 | T2N3M0 | IIIA | Diffuse | Poor |
| 245 | | M | 74 | T3N1M0 | IIB | Intestinal | Moderate |
| 246 | | F | 52 | T3N2M0 | IIIA | Intestinal | Moderate |
| 247 | | M | 57 | T3N1M0 | IIB | Mixed | Poor |
| 248 | | M | 62 | T3N3M0 | IIIB | Mixed | Poor |
| 249 | | M | 69 | T3N0M0 | IIA | Intestinal | Moderate |
| 250 | | M | 62 | T3N3M0 | IIIB | Diffuse | Poor |
| 251 | | F | 79 | T2N0M0 | IB | Intestinal | Poor |
| 252 | | M | 58 | T3N3M0 | IIIB | Mixed | Poor |
| 253 | | M | 36 | T2N2M0 | IIB | Diffuse | Poor |
| 254 | | M | 46 | T3N3M0 | IIIB | Diffuse | Poor |
| 255 | | M | 52 | T3N0M0 | IIA | Intestinal | Moderate |
| 256 | | M | 60 | T4aN2M0 | IIIB | N/A | Moderate |
| 257 | | F | 71 | T3N2M0 | IIIA | Mixed | Moderate |
| 258 | | F | 81 | T4bN3M0 | IIIC | Mixed | Moderate |
| 259 | | F | 46 | T3N2M0 | IIIA | Diffuse | Poor |
| 260 | | M | 74 | T2N1M0 | IIA | Intestinal | Moderate |
| 261 | | F | 62 | T2N3M0 | IIIA | Diffuse | Poor |
| 262 | | M | 47 | T2N0M0 | IB | Diffuse | Poor |
| 263 | | F | 71 | T2N0M0 | IB | Mixed | Poor |
| 264 | | M | 69 | T3N0M0 | IIA | Diffuse | Poor |
| 265 | | M | 57 | T3N2M0 | IIIA | Intestinal | Moderate |
| 266 | | M | 63 | T3N3M0 | IIIB | Intestinal | Moderate |
| 267 | | M | 57 | T3N3M0 | IIIB | Mixed | Moderate |
| 268 | | M | 50 | T3N1M0 | IIB | Mixed | Moderate |
| 269 | | M | 64 | T4bN2M0 | IIIC | Intestinal | Moderate |
| 270 | | M | 71 | T2N0M0 | IB | Mixed | Poor |
| 271 | | M | 33 | T3N2M0 | IIIA | Mixed | Poor |
| 272 | | M | 69 | T3N0M0 | IIA | Intestinal | Moderate |
| 273 | | M | 56 | T3N3M0 | IIIB | Intestinal | Moderate |
| 274 | | M | 42 | T3N0M0 | IIA | Mixed | Moderate |
| 275 | | M | 52 | T3N3M0 | IIIB | Mixed | Moderate |
| 276 | | F | 64 | T3N3M0 | IIIB | Intestinal | Moderate |
| 277 | | F | 60 | T3N1M0 | IIB | N/A | Moderate |
| 278 | | M | 56 | T3N3M0 | IIIB | N/A | Moderate |
| 279 | | M | 66 | T3N3M0 | IIIB | Diffuse | Poor |
| 280 | | M | 51 | T3N3M0 | IIIB | Intestinal | Moderate |
| 281 | | F | 47 | T3N2M0 | IIIA | N/A | Moderate |
| 282 | | M | 59 | T3N3M0 | IIIB | Intestinal | Moderate |

**Table S2.** List of the nucleic acid sequences used for molecular cloning experiments.

| Name | Seqenuces (5'→3') |
| --- | --- |
| Flag-USP15-WT CDS | NM_001252078.2, AATACGTGTTTCATG (886-900) & CCGAATTGTAAAGAA (2428-2442) |
| USP15-298A CDS | NM_001252078.2, AATACGGCTTTCATG (886-900) |
| USP15-812A CDS | NM_001252078.2, CCGAATGCTAAAGAA (2428-2442) |
| His-HKDC1-WT CDS | NM_025130.4 |
| USP15-shRNA-1# | CTAGTTCATGTGCATTAGGCTGCCGTATATACTACTATGAT |
| USP15-shRNA-2# | TAGGACAGAAGATACAGAGCACGTGATTATTCCTGTTTGCC |
| HKDC1-siRNA-1# | CAGTTCTACCCAACGCCCAATGAAA |
| HKDC1-siRNA-2# | CGGCACAGAGCTGTTTGAATATGTA |
| HKDC1 blocker | A short competitive peptide, HAIYPRHLQQAQKEN |

**Table S3.** List of the primers used for qPCR analyses.

| Gene_name | qPCR primers | Seqenuces (5'→3') |
| --- | --- | --- |
| GLUT1 | Forward | CATCCCATGGTTCATCGTGGCTGAACT |
|  | Reverse | GAAGTAGGTGAAGATGAAGAACAGAAC |
| SLC2A4 (GLUT4) | Forward | TGGGCGGCATGATTTCCTC |
|  | Reverse | GCCAGGACATTGTTGACCAG |
| HK1 | Forward | GCTCTCCGATGAAACTCTCATAG |
|  | Reverse | GGACCTTACGAATGTTGGCAA |
| HK2 | Forward | GAGCCACCACTCACCCTACT |
|  | Reverse | CCAGGCATTCGGCAATGTG |
| HK3 | Forward | GGACAGGAGCACCCTCATTTC |
|  | Reverse | CCTCCGAATGGCATCTCTCAG |
| GCK (HK4) | Forward | CCTGGGTGGCACTAACTTCAG |
|  | Reverse | TAGTCGAAGAGCATCTCAGCA |
| HKDC1 | Forward | GTTGCCCACCTTCGTCAGG |
|  | Reverse | AGCGACTTGCACCTTCAGC |
| IGFBP1 | Forward | TTGGGACGCCATCAGTACCTA |
|  | Reverse | TTGGCTAAACTCTCTACGACTCT |
| IGFBP2 | Forward | GACAATGGCGATGACCACTCA |
|  | Reverse | CAGCTCCTTCATACCCGACTT |
| IGFBP3 | Forward | AGAGCACAGATACCCAGAACT |
|  | Reverse | GGTGATTCAGTGTGTCTTCCATT |
| IGF2BP1 | Forward | GCGGCCAGTTCTTGGTCAA |
|  | Reverse | TTGGGCACCGAATGTTCAATC |
| IGF2BP2 | Forward | AGTGGAATTGCATGGGAAAATCA |
|  | Reverse | CAACGGCGGTTTCTGTGTC |
| IGF2BP3 | Forward | TATATCGGAAACCTCAGCGAGA |
|  | Reverse | GGACCGAGTGCTCAACTTCT |
| GPI | Forward | CAAGGACCGCTTCAACCACTT |
|  | Reverse | CCAGGATGGGTGTGTTTGACC |
| PFKL | Forward | GCTGGGCGGCACTATCATT |
|  | Reverse | TCAGGTGCGAGTAGGTCCG |
| ALDOA | Forward | ATGCCCTACCAATATCCAGCA |
|  | Reverse | GCTCCCAGTGGACTCATCTG |
| PGK1 | Forward | TGGACGTTAAAGGGAAGCGG |
|  | Reverse | GCTCATAAGGACTACCGACTTGG |
| PGAM1 | Forward | TCTGGAGGCGCTCCTATGAT |
|  | Reverse | TCTGTGAGGTCTGCATACCTG |
| ENO1 | Forward | TGGTGTCTATCGAAGATCCCTT |
|  | Reverse | CCTTGGCGATCCTCTTTGG |
| PKM2 | Forward | ATAACGCCTACATGGAAAAGTGT |
|  | Reverse | TAAGCCCATCATCCACGTAGA |
| LDHA | Forward | ATGGCAACTCTAAAGGATCAGC |
|  | Reverse | CCAACCCCAACAACTGTAATCT |
| PDK1 | Forward | CTGTGATACGGATCAGAAACCG |
|  | Reverse | TCCACCAAACAATAAAGAGTGCT |
| TUBB | Forward | AAGATCCGAGAAGAATACCCTGA |
|  | Reverse | CTACCAACTGATGGACGGAGA |

**Table S4.** LC-MS/MS analysis of the Co-immunoprecipitation of USP15 (Top 15).

| No. | Accession | Gene symbol | Score | Coverage | # Unique Peptides | # Peptides | # PSMs | MW [kDa] |
| --- | --- | --- | --- | --- | --- | --- | --- | --- |
| 1 | Q9Y4E8 | USP15 | 404.8265 | 35.47 | 34 | 34 | 171 | 112.3478 |
| 2 | Q15020 | SART3 | 245.2082 | 22.85 | 19 | 19 | 79 | 109.8653 |
| 3 | P38646 | HSPA9 | 162.1527 | 36.82 | 23 | 23 | 60 | 73.63478 |
| 4 | P78527 | PRKDC | 153.6755 | 8.65 | 30 | 30 | 61 | 468.7879 |
| 5 | P11142 | HSPA8 | 152.1089 | 41.95 | 14 | 19 | 55 | 70.85423 |
| 6 | P04114 | APOB | 145.3807 | 10.56 | 24 | 24 | 57 | 515.2829 |
| 7 | P09874 | PARP1 | 132.5775 | 17.95 | 16 | 16 | 47 | 113.0124 |
| 8 | Q92841 | DDX17 | 124.0445 | 30.32 | 14 | 21 | 50 | 80.22202 |
| 9 | P17844 | DDX5 | 120.8139 | 32.08 | 15 | 22 | 47 | 69.10474 |
| 10 | P15328 | FOLR1 | 111.1165 | 24.51 | 6 | 6 | 33 | 29.79922 |
| 11 | P05187 | ALPP | 75.04323 | 37.76 | 5 | 16 | 31 | 57.91729 |
| 12 | P31327 | CPS1 | 74.45721 | 11.8 | 16 | 16 | 29 | 164.8348 |
| 13 | O00425 | IGF2BP3 | 55.58225 | 29.53 | 11 | 14 | 22 | 63.66556 |
| 14 | Q9Y3F4 | STRAP | 47.8888 | 24 | 6 | 6 | 15 | 38.41398 |
| 15 | Q2TB90 | HKDC1 | 47.76609 | 17.34 | 14 | 14 | 19 | 102.4785 |
